# Analysis Tools for Interconnected Boolean Networks With Biological Applications

**DOI:** 10.3389/fphys.2018.00586

**Published:** 2018-05-29

**Authors:** Madalena Chaves, Laurent Tournier

**Affiliations:** ^1^Inria Sophia Antipolis - Méditerranée, Université Côte d'Azur, Valbonne, France; ^2^MaIAGE, INRA, Université Paris-Saclay, Jouy-en-Josas, France

**Keywords:** asynchronous Boolean networks, module interconnection, state transition graph, attractor computation, biological regulatory networks

## Abstract

Boolean networks with asynchronous updates are a class of logical models particularly well adapted to describe the dynamics of biological networks with uncertain measures. The state space of these models can be described by an asynchronous state transition graph, which represents all the possible exits from every single state, and gives a global image of all the possible trajectories of the system. In addition, the asynchronous state transition graph can be associated with an absorbing Markov chain, further providing a semi-quantitative framework where it becomes possible to compute probabilities for the different trajectories. For large networks, however, such direct analyses become computationally untractable, given the exponential dimension of the graph. Exploiting the general modularity of biological systems, we have introduced the novel concept of *asymptotic graph*, computed as an interconnection of several asynchronous transition graphs and recovering all asymptotic behaviors of a large interconnected system from the behavior of its smaller modules. From a modeling point of view, the interconnection of networks is very useful to address for instance the interplay between known biological modules and to test different hypotheses on the nature of their mutual regulatory links. This paper develops two new features of this general methodology: a quantitative dimension is added to the asymptotic graph, through the computation of relative probabilities for each final attractor and a companion *cross-graph* is introduced to complement the method on a theoretical point of view.

## 1. Introduction

An intuitive representation of system interactions, an algorithmic description of state transitions, and the capacity to capture the global dynamics of the system, list some of the advantages of Boolean models, which remain a powerful tool in the modeling and analysis of biological networks (Wang et al., 2012; Abou-Jaoudé et al., 2016). Successfully predictive examples of Boolean models cover complex networks across many different organisms, from cell cycle (Li et al., 2004; Fauré et al., 2006), to fly or plant morphogenesis (Albert and Othmer, 2003; García-Gómez et al., 2017), and highly complex networks such as T-cell induction (Mendoza and Xenarios, 2006; Saez-Rodriguez et al., 2007), leukemia (Zhang et al., 2008) or apoptosis (Calzone et al., 2010).

In a modular view of a biological organism, each task is executed by a specific set of interactions among an ensemble of biological components; in other words, it can be said that there is a specifc network, or module, for each specific task (signaling, metabolic, physiological, etc.). These modules often interact with each other, one task triggering the next in a chain of events or cyclic phenomena. Examples include chains of signaling networks such as MAPK cascades, genetic-metabolic interactions (Baldazzi et al., 2010), or coupled oscillations (Gérard and Goldbeter, 2012). However, in many cases, while experimental evidence supports the existence of links between two modules, their modes of interaction are still unclear (as in the case of mammalian cell cycle and circadian clock, see Feillet et al., 2015). In this context, mathematical tools are necessary to facilitate the analysis of the complex behavior obtained from the interconnection of two or more known modules.

One of the challenges in the analysis of Boolean networks is attractor computation, particularly for high-dimensional networks. For a network of dimension *n*, the size of the state transition graph is 2^*n*^. A direct analysis of such a graph may become computationally costly, in terms of space and time, when *n* ≥ 20. This is especially true with asynchronous updating, which includes numerous dynamical trajectories. Two very efficient methods have recently been developed: Zañudo and Albert (2013) compute all attractors of a network (up to *n* ≈ 100), by isolating special properties of the state transition graph's components; Veliz-Cuba et al. (2014) compute all singletons (attractors containing a single state) for networks up to *n* = 1,000, by using a computational algebra approach.

In this paper, we propose a methodology aimed specifically at analyzing the interconnection between several known Boolean modules. The interconnection between two biological networks can be very hard to test *in vivo*: our methodology provides a platform for hypothesis testing, confirming or disproving assumptions regarding mutual regulatory effects, simulating and comparing various forms of interconnection schemes and corresponding emergent dynamical behavior. Our method relies on the construction of a new object, *the asymptotic graph*, introduced by Tournier and Chaves (2013), which is a directed graph constructed only from the set of attractors of each module and that captures all the asymptotic behaviors of the interconnected network.

After a brief review of Boolean network interconnections, two improvements to the asymptotic graph are introduced in this paper, to mitigate two of its known limitations. First, it was observed that the asymptotic graph may also recover spurious attractors, in addition to the true attractors of the full network (Tournier and Chaves, 2013); we introduce an extension, called the *cross graph* that solves this issue from a theoretical point of view. The cross graph is constructed from the set of strongly connected components of each separate module, while the asymptotic graph is constructed from *terminal* strongly connected components only. Second, to enrich the traditional ON/OFF representation inherent to Boolean models, we propose a method to assign probabilities to the edges of the asymptotic graph, thereby allowing a probabilistic representation of the various possible trajectories of the composed network. Our methodology is applied first to a class of general randomly generated Boolean models and then to two state-of-the-art biological models in two different organisms: (i) to explore the interplay between mammalian cell cycle and circadian clock oscillators and (ii) to test hypotheses on the regulatory links between budding yeast cell cycle and cell size, where our analysis suggests that the START signal should come from mitosis phase.

## 2. Interconnections of asynchronous boolean networks: a short review

Throughout this paper, we will consider Boolean networks under asynchronous updates. An interconnected Boolean network is, briefly, the combined network formed by linking together, in an approriately prescribed way, two or more separate Boolean modules. In previous works (Chaves and Tournier, 2011; Tournier and Chaves, 2013) we have introduced a new object, the *asymptotic graph*, that characterizes the attractors of the combined Boolean network in terms only of the attractors of the separate modules—hence with no need to compute the larger state transition graph. In the following, the definition of the main objects needed to introduce the asymptotic graph are briefly reviewed.

### 2.1. IO asynchronous boolean networks and their interconnections

Let us start by a brief recall of the definition of an input-output asynchronous Boolean network (IO ABN), reprising the notation introduced by Tournier and Chaves (2013). An IO ABN Σ^*A*^ is characterized by three integers *n*_*A*_, *p*_*A*_, *q*_*A*_ (*n*_*A*_ > 0 is the dimension of the system, *p*_*A*_, *q*_*A*_ ≥ 0 are respectively the numbers of inputs and outputs) and by two Boolean maps: $f^{A}:{\left\{0,1\right\}}^{p_{A}}\times {\left\{0,1\right\}}^{n_{A}}\to {\left\{0,1\right\}}^{n_{A}}$ (the transition function) and $h^{A}:{\left\{0,1\right\}}^{n_{A}}\to {\left\{0,1\right\}}^{q_{A}}$ (the output function). For any given input profile $u\in {\left\{0,1\right\}}^{p_{A}}$, the asynchronous dynamics of the network are given by the *asynchronous transition graph* *G*^*A, u*^, which is a digraph over the vertex set ${\left\{0,1\right\}}^{n_{A}}$ defined as follows: for any state $x=\left(x_{1},\ldots ,x_{n}\right)\in {\left\{0,1\right\}}^{n_{A}}$, the set of its successors are the states (*x*_1_, …, ¬*x*_*i*_, …, *x*_*n*_), for all *i* ∈ {1, …, *n*} such that $f_{i}^{A}\left(u,x\right)\neq x_{i}$. The number of vertices of such a graph is $2^{n_{A}}$ and its number of arcs, denoted by *m*_*A*_, verifies $0\leq m_{A}\leq n_{A}2^{n_{A}}$. It is therefore relatively sparse and can thus be efficiently stored by a $2^{n_{A}}\times 2^{n_{A}}$ adjacency matrix. In the following, we will consider that *G*^*A, u*^ designates this matrix. Given two integers $i,j\in \left\{1,\ldots ,2^{n_{A}}\right\}$, the (*i, j*) entry of the adjacency matrix equals 1 if state *j* is a successor of state *i* and 0 otherwise. In a classical abuse of notation, we associate each integer $i\in \left\{1,\ldots ,2^{n_{A}}\right\}$ with its binary representation $x\in {\left\{0,1\right\}}^{n_{A}}$ in lexicographic order, with the left-most bit being the most significant one; in other words: $i-1=\overset{n_{A}}{\underset{k=1}{\sum }}x_{k}2^{n_{A}-k}$. Thus, we will indifferently call *state* either an integer $i\in \left\{1,\ldots ,2^{n_{A}}\right\}$ or its Boolean representation $x\in {\left\{0,1\right\}}^{n_{A}}$.

example 1. Consider the bidimensional single-input, single-output (SISO) network defined by: $f^{A}\left(u,x_{1},x_{2}\right)=\left(u,x_{1}\right)$ and $h^{A}\left(x_{1},x_{2}\right)=x_{2}$. Graphically, this network can be represented as a simple cascade *u* → *x*_1_ → *x*_2_. Its dynamics are characterized by the two graphs *G*^*A*,0^ and *G*^*A*, 1^, represented below in graphical and matricial forms:

$$\begin{array}{l} G^{A,0}:\;\begin{array}{ccc} 10 & \to & 11\\ ↓ & \; & ↓\\ 00 & \leftarrow & 01 \end{array}\;\left(\begin{array}{cccc} 0 & 0 & 0 & 0\\ 1 & 0 & 0 & 0\\ 1 & 0 & 0 & 1\\ 0 & 1 & 0 & 0 \end{array}\right),\\ G^{A,1}:\;\begin{array}{ccc} 01 & \to & 00\\ ↓ & \; & ↓\\ 11 & \leftarrow & 10 \end{array}\;\left(\begin{array}{cccc} 0 & 0 & 1 & 0\\ 1 & 0 & 0 & 1\\ 0 & 0 & 0 & 1\\ 0 & 0 & 0 & 0 \end{array}\right). \end{array}$$

In adjacency matrices, by convention the (*i, j*) entry equals 1 iff state *j* is a successor of state *i*. Here, the four states (rows and columns of the matrix) are intended in the following order: 00, 01, 10, 11. In *G*^*A*,0^, state 00 does not have any successor, implying the first row of its adjacency matrix is zero: 00 is a steady state of the network. Similarly, 11 is a steady state of *G*^*A*,1^.    □

Classically, an asynchronous transition graph *G*^*A, u*^ is analyzed by first computing its decomposition into strongly connected components (SCCs), denoted by $A_{u}^{1},\ldots ,A_{u}^{N_{u}^{A}}$, where $1\leq N_{u}^{A}\leq 2^{n}$. The set of all SCCs forms a partition of the state space ${\left\{0,1\right\}}^{n_{A}}$ and their computation can be efficiently achieved in $O\left(2^{n_{A}}+m_{A}\right)$. By contracting each SCC to a single vertex, a directed acyclic graph (dag) is constructed, sometimes called *condensation* graph or simply SCC graph. This dag provides a useful description of key dynamical behaviors of the network; in particular terminal SCCs (the leafs of the dag) correspond to the attractors of the network. More details about these graph theoretical tools can be found, for instance, in the textbook by Cormen et al. (2001).

Consider now two IO ABN Σ^*A*^ and Σ^*B*^, of respective dimensions (*n*_*A*_, *p*_*A*_, *q*_*A*_) and (*n*_*B*_, *p*_*B*_, *q*_*B*_) and state variables $x\in {\left\{0,1\right\}}^{n_{A}}$ and $y\in {\left\{0,1\right\}}^{n_{B}}$. Note that all the methods presented in this paper generalize to more than two modules; however, in order to maintain a clear exposition of the results, the definitions are given for interconnections of two modules. An *interconnection scheme* of Σ^*A*^ and Σ^*B*^ consists in two interconnecting functions $\mu_{A}:{\left\{0,1\right\}}^{q_{B}}\to {\left\{0,1\right\}}^{p_{A}}$ and $\mu_{B}:{\left\{0,1\right\}}^{q_{A}}\to {\left\{0,1\right\}}^{p_{B}}$ mapping the outputs of each module to the inputs of the other module. For convenience, throughout this paper we will make the assumption that *q*_*B*_ = *p*_*A*_ and *q*_*A*_ = *p*_*B*_ and that the interconnecting functions are simply identity maps. Following Tournier and Chaves (2013), with this assumption the resulting interconnected network is the ABN of dimension *n*_*A*_ + *n*_*B*_, with no input and no output, defined by the following transition function:

(1) $$\begin{array}{c} f:{\left\{0,1\right\}}^{n_{A}}\times \;{\left\{0,1\right\}}^{n_{B}}\to \;{\left\{0,1\right\}}^{n_{A}}\times \;{\left\{0,1\right\}}^{n_{B}}\\ \;\left(x,y\right)\;\mapsto \;(f^{A}(h^{B}(y),x),f^{B}(h^{A}(x),y)). \end{array}$$

One can then consider the interconnection as a standalone network: its transition graph *G* can be constructed from this transition function *f*. Alternatively, one can also build the graph *G* directly from the set of transition graphs $G^{A,u},u\in {\left\{0,1\right\}}^{p_{A}}$ and $G^{B,\upsilon },\upsilon \in {\left\{0,1\right\}}^{p_{B}}$ as follows. Let (*x, y*) and (*x*′, *y*′) be two Boolean vectors in ${\left\{0,1\right\}}^{n_{A}}\times {\left\{0,1\right\}}^{n_{B}}$, then (*x*′, *y*′) is a (asynchronous) successor of (*x, y*) if

- either *x* = *x*′ and *y*′ is a successor of *y* in *G*^*B*,*h*^*A*^(*x*)^,
- or *y* = *y*′ and *x*′ is a successor of *x* in *G*^*A*,*h*^*B*^(*y*)^.

It is possible to summarize this definition in a simple matricial form. First, for each $\alpha \in {\left\{0,1\right\}}^{q_{A}}$, introduce the $2^{n_{A}}\times 2^{n_{A}}$ diagonal Boolean matrix Δ^*A*,α^ such that ${\left[\Delta^{A,\alpha }\right]}_{ii}=1$ if the output of state *i* is equal to α and 0 otherwise. Similarly, for module Σ^*B*^ introduce the $2^{n_{B}}\times 2^{n_{B}}$ diagonal Boolean matrices Δ^*B*,β^, with $\beta \in {\left\{0,1\right\}}^{q_{B}}$. Then, *G* can be reconstructed by the formula:

(2) $$\begin{array}{c} G:=\underset{(\alpha ,\beta )\in {\left\{0,1\right\}}^{q_{A}}\times \;{\left\{0,1\right\}}^{q_{B}}}{\vee }\left(G^{A,\beta }⊗\Delta^{B,\beta }\vee \Delta^{A,\alpha }⊗G^{B,\alpha }\right), \end{array}$$

where ⊗ designates the classical Kronecker product. By replacing matrices Δ with identity matrices, one may recognize in this definition of *G* the notion of Cartesian product of graphs, first introduced by Sabidussi (1959). To be more precise, (2) generalizes the notion of Cartesian product to interconnections, by including only transitions that are consistent with the input-output scheme.

example 2. Consider module Σ^*A*^ defined in Example 1 and let the one-dimensional SISO module Σ^*B*^ defined by $f^{B}\left(\upsilon ,y_{1}\right)=\neg \upsilon$ and $h^{B}\left(y_{1}\right)=y_{1}$. Its dynamics are given by

$$G^{B,0}=\left(\begin{array}{cc} 0 & 1\\ 0 & 0 \end{array}\right),\text{ and }G^{B,1}=\left(\begin{array}{cc} 0 & 0\\ 1 & 0 \end{array}\right).$$

The interconnected network can be reconstructed by using (1), leading to the 3-dimensional transition function *f*(*x*_1_, *x*_2_, *y*_1_) = (*y*_1_, *x*_1_, ¬*x*_2_). Alternatively, the transition graph *G* can also be computed directly as the interconnection of the dynamics of the two separated modules by using (2):

$$\begin{array}{l} G=\left(G^{A,0}⊗\Delta^{B,0}\right)\vee \left(G^{A,1}⊗\Delta^{B,1}\right)\vee \left(\Delta^{A,0}⊗G^{B,0}\right)\vee \left(\Delta^{A,1}⊗G^{B,1}\right),\\ \;=\left(\begin{array}{cccc} 0 & 0 & 0 & 0\\ 1 & 0 & 0 & 0\\ 1 & 0 & 0 & 1\\ 0 & 1 & 0 & 0 \end{array}\right)⊗\left(\begin{array}{cc} 0 & 0\\ 0 & 1 \end{array}\right)\vee \left(\begin{array}{cccc} 0 & 0 & 1 & 0\\ 1 & 0 & 0 & 1\\ 0 & 0 & 0 & 1\\ 0 & 0 & 0 & 0 \end{array}\right)⊗\left(\begin{array}{cc} 1 & 0\\ 0 & 0 \end{array}\right)\vee \\ \;\;\;\;\;\;\left(\begin{array}{cccc} 1 & 0 & 0 & 0\\ 0 & 0 & 0 & 0\\ 0 & 0 & 1 & 0\\ 0 & 0 & 0 & 0 \end{array}\right)⊗\left(\begin{array}{cc} 0 & 1\\ 0 & 0 \end{array}\right)\vee \left(\begin{array}{cccc} 0 & 0 & 0 & 0\\ 0 & 1 & 0 & 0\\ 0 & 0 & 0 & 0\\ 0 & 0 & 0 & 1 \end{array}\right)⊗\left(\begin{array}{cc} 0 & 0\\ 1 & 0 \end{array}\right),\\ \;\;=\left(\begin{array}{cccccccc} 0 & 1 & 0 & 0 & 0 & 0 & 0 & 0\\ 0 & 0 & 0 & 0 & 0 & 1 & 0 & 0\\ 1 & 0 & 0 & 0 & 0 & 0 & 0 & 0\\ 0 & 1 & 1 & 0 & 0 & 0 & 0 & 1\\ 1 & 0 & 0 & 0 & 0 & 1 & 1 & 0\\ 0 & 0 & 0 & 0 & 0 & 0 & 0 & 1\\ 0 & 0 & 1 & 0 & 0 & 0 & 0 & 0\\ 0 & 0 & 0 & 0 & 0 & 0 & 1 & 0 \end{array}\right). \end{array}$$

In graphical form, this transition graph *G* of the interconnected network can be represented as:
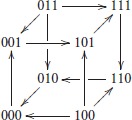


This graph has a unique attractor, composed of six states: {001, 101, 111, 110, 010, 000}.    □

In the present paper, note that we assume the modules and the interconnection scheme are given. It is also possible to consider interconnections as a general *model reduction* technique, where a large network is first decomposed into *a priori* unknown sub-networks. The identification of an efficient decomposition, with the corresponding interconnecting scheme, would then become critical. This problem is related to the general problem of graph partitioning and is addressed elsewhere (Tournier and Chaves, 2013).

### 2.2. The asymptotic graph of an interconnection

We can now give the definition of the asymptotic graph (Tournier and Chaves, 2013). First, list all the terminal SCCs of module Σ^*A*^: $\left\{A_{u}^{i},u\in {\left\{0,1\right\}}^{p_{A}},1\leq i\leq L_{u}^{A}\right\}$ and cut them with respect to their outputs, *ie*. define, for each output profile $\alpha \in {\left\{0,1\right\}}^{q_{A}}$, the set $A_{u\alpha }^{i}:=\left\{x\in A_{u}^{i},h^{A}\left(x\right)=\alpha \right\}$. For some α such a set may be empty, in that case we will simply omit it. Similarly, define $\left\{B_{\upsilon \beta }^{j},\upsilon \in {\text{\{0,1\}}}^{p_{B}},\alpha \in {\text{\{0,1\}}}^{q_{B}},1\leq j\leq L_{v}^{B}\right\}$ for module Σ^*B*^. The *asymptotic graph* of the interconnection is then defined as the directed graph *G*^*as*^ = (*V*^*as*^, *E*^*as*^) such that the vertex set *V*^*as*^ is composed of all the cross products $A_{u\alpha }^{i}\times B_{\upsilon \beta }^{j}$ and the arc set *E*^*as*^ is constructed as follows:

- $A_{u\alpha }^{i}\times B_{v\beta }^{j}\to A_{\beta \alpha^{\prime }}^{i^{\prime }}\times B_{v\beta }^{j}$ iff there exist $x\in A_{u\alpha }^{i}$, $x^{\prime }\in A_{\beta \alpha^{\prime }}^{i^{\prime }}$ such that there exists a path from *x* to *x*′ in *G*^*A*,β^,
- $A_{u\alpha }^{i}\times B_{v\beta }^{j}\to A_{u\alpha }^{i}\times B_{\alpha \beta^{\prime }}^{j^{\prime }}$ iff there exist $y\in B_{v\beta }^{j}$, $y^{\prime }\in B_{\alpha \beta^{\prime }}^{j^{\prime }}$ such that there exists a path from *y* to *y*′ in *G*^*B*,α^.

Finally, introduce the function π as follows: if $V=A_{u\alpha }^{i}\times B_{\upsilon \beta }^{j}\in V^{as},\pi (V):=\{(x,y),x\in A_{u\alpha }^{i},y\in B_{v\beta }^{j}\}$, $\pi \left(V\right):=\left\{\left(x,y\right),x\in A_{u\alpha }^{i},y\in B_{v\beta }^{j}\right\}$ and if *R*⊆*V*^*as*^, π(*R*): = ⋃_*V* ∈ *R*_ π(*V*). The interest of the asymptotic graph lies in the following theorem, a proof of which can be found in Tournier and Chaves (2013).

theorem 1. *If* *Q* *is an attractor of the interconnected network, then there exists a terminal SCC* *R* *of* *G*^*as*^ *such that* π(*R*) ⊆ *Q*.

example 3. Consider the interconnection of Example 2 above. The asymptotic graph is given by

$$\begin{array}{ccc} A_{00}^{1}\times B_{01}^{1} & \to & A_{11}^{1}\times B_{01}^{1}\\ ↑ & \; & ↓\\ A_{00}^{1}\times B_{10}^{1} & \to & A_{11}^{1}\times B_{10}^{1} \end{array}\;\text{ with: }\;\{\begin{array}{lll} \pi (A_{00}^{1}\times B_{01}^{1}) & = & \{001\},\\ \pi (A_{11}^{1}\times B_{01}^{1}) & = & \{111\},\\ \pi (A_{11}^{1}\times B_{10}^{1}) & = & \{000\},\\ \pi (A_{00}^{1}\times B_{10}^{1}) & = & \{110\}. \end{array}$$

Therefore, *G^as^* is composed of a single terminal SCC *R*, and π(*R*) = {001, 111, 000, 110} is actually included into the (unique) attractor of the interconnected network.    □

Thanks to Theorem 1, the asymptotic graph is a powerful analytic tool as it recovers *all* the attractors of an interconnection (without missing any), by constructing a graph significantly smaller than the full interconnected graph *G* (section 4 below provides numerical results for random interconnections). However, it may happen that some terminal SCC of *G^as^* does not correspond to an actual attractor of the interconnection. Such terminal SCCs, called *spurious* attractors, appear very rarely and there exist some sufficient conditions to detect *a priori* spurious attractors in certain cases. The most simple one, particularly useful for biological applications is the fact that when *R* is a singleton then it cannot be a spurious attractor. The proof, along with additional conditions are provided elsewhere (Tournier and Chaves, 2013; Chaves and Carta, 2015).

## 3. New analysis tools

This section describes our new contributions. Our first goal is to improve the asymptotic graph construction to avoid the generation of spurious attractors (section 3.1) and our second goal is to update the asymptotic graph by adding quantitative information (probabilistic) on the state transitions (section 3.2).

### 3.1. A theoretical tool to recover all the dynamics of an interconnection

The asymptotic graph of an interconnection is constructed only from the modules' attractors, generally implying a relatively manageable size allowing to analyze a wide range of practical examples of interconnections (see sections 4 and 5). Nevertheless, ignoring transient dynamical behaviors of the modules also implies two drawbacks for Theorem 1. First, spurious attractors may appear, although this phenomenon seems to be relatively rare as illustrated in section 4. Second, when a terminal SCC of *G^as^* corresponds to an actual attractor, Theorem 1 only ensures an inclusion, meaning the predicted attractor may contain only a small proportion of states that are in the real attractor. We now propose a new graph, called the *cross-graph*, overcoming those two issues and ensuring, at the price of a higher computational cost, a one-to-one recovery of all the attractors of the interconnected network. Note that Tournier and Chaves (2013) already introduced a notion of cross-graph, however the cross-graph described in the following is significantly improved. In particular, its size is bounded by the size of the full interconnected graph, which was not the case for the older version.

Let Σ*^A^* and Σ*^B^* be two IO ABN of respective dimensions (*n_A_*, *p_A_*, *q_A_*) and (*n_B_*, *p_B_*, *q_B_*). As before, suppose for convenience that *p_A_* = *q_B_*, *p_B_* = *q_A_* and the interconnecting maps are simply identity maps. We also assume that each module has been separately analyzed: the transition graphs *G^A,u^*, *u* ∈ {0, 1}^*p_A_*^ and *G^B,υ^*, υ ∈ {0, 1}^*p_B_*^ have been constructed and decomposed into strongly connected components $\left\{A_{u}^{i},1\leq i\leq N_{u}^{A}\right\}$ for each *u* ∈ {0, 1}^*p_A_*^ and $\left\{B_{\upsilon }^{j},1\leq j\leq N_{u}^{B}\right\}$ for each υ ∈ {0, 1}^*p_B_*^. Let *G* denote the full transition graph of the interconnected network, of size 2^*n_A_*+*n_B_*^. It can be computed thanks to (2), by interconnecting the modules' transition graphs. The idea behind the cross-graph is to generalize formula (2) in order to interconnect directly the SCCs of those graphs instead of the whole graphs themselves, thus potentially saving a significant amount of space when constructing the dynamics of the interconnection.

First, observe that the strongly connected components $\left\{A_{u}^{i},1\leq i\leq N_{u}^{A}\right\}$ form a partition of the state space {0, 1}^*n_A_*^ of module Σ*^A^* ($N_{u}^{A}$ are integers verifying $1\leq N_{u}^{A}\leq 2^{n_{A}}$). Therefore, for *u* varying in {0, 1}^*p_A_*^ we obtain 2^*p_A_*^ partitions of the same finite set Ω = {0, 1}^*n_A_*^. Let 𝔓_Ω_ denote the set of all partitions of Ω. Given two partitions *P*_1_, *P*_2_ ∈ 𝔓_Ω_, *P*_1_ is said *finer than* *P*_2_, denoted by *P*_1_ ≤ *P*_2_ if, for each element *p* in *P*_1_ there is an element *q* in *P*_2_ such that *p* ⊆ *q* (in other words, partition *P*_1_ is a *fragmentation* of partition *P*_2_). The set (𝔓_Ω_, ≤) has the structure of a geometric lattice (see *eg*. Birkhoff, 1940). Consequently, for any set *S* ⊆ 𝔓_Ω_, there exists a (unique) greatest lower bound of *S* denoted by ∧ *S* ∈ 𝔓_Ω_. Coming back to the SCC decompositions, introduce the following partition:

$$\begin{array}{l} Z^{A}\;:=\underset{u\in {\left\{0,1\right\}}^{p_{A}}}{\wedge }\left\{A_{u}^{i},1\leq i\leq N_{u}^{A}\right\},\\ \;\;\;\;\;\;\;\;\;\;=\left\{A^{1},\ldots ,A^{N^{A}}\right\}, \end{array}$$

which is the coarsest partition of {0, 1}^*n_A_*^ that is finer than every SCC decomposition of all the transition graphs *G^A,u^*. Once this partition is constructed, following the same idea as before it is further refined by cutting each set **A**^*i*^ according to their outputs: $A_{\alpha }^{i}:=\left\{x\in A^{i},h^{A}(x)=\alpha \right\}$, with the convention that such sets are simply omitted when they are empty. Therefore, we finally obtain a partition $Z_{h}^{A}=\left\{A_{\alpha }^{i},1\leq i\leq N^{A},\alpha \in {\text{\{0,1\}}}^{q_{A}}\right\}$ of the state space {0, 1}^*n_A_*^ that is compatible with every SCC decompositions of the dynamics of modules Σ*^A^*. By construction, the number of elements in this partition, denoted by *M_A_*, verifies 1 ≤ *M_A_* ≤ 2^*n_A_*^. Applying the exact same procedure for module Σ*^B^*, one obtains a similar partition $Z_{h}^{B}=\left\{B_{\beta }^{j},1\leq j\leq N^{B},\beta \in {\left\{0,1\right\}}^{q_{B}}\right\}$ of the state space {0, 1}^*n_B_*^, containing *M_B_* elements.

Once partitions $Z_{h}^{A}$ and $Z_{h}^{B}$ are defined, the construction of the cross graph closely resembles the one of the asymptotic graph. The cross graph is the digraph *G^cr^* = (*V^cr^*, *E^cr^*), where the vertex set *V^cr^* is composed of all cross-products $A_{\alpha }^{i}$ × $B_{\beta }^{j}$ and the arc set is constructed as follows:

- $A_{\alpha }^{i}\times B_{\beta }^{j}\to A_{\alpha^{\prime }}^{i^{\prime }}\times B_{\beta }^{j}$ iff there exist *a* ∈ $A_{\alpha }^{i}$, $a\in A_{\alpha }^{i},a^{\prime }\in A_{\alpha^{\prime }}^{i^{\prime }}$ such that there is a transition from *a* to *a′* in graph *G^A,β^*,
- $A_{\alpha }^{i}\times B_{\beta }^{j}\to A_{\alpha }^{i}\times B_{\beta^{\prime }}^{j^{\prime }}$ iff there exist *b* ∈ $B_{\beta }^{j}$, $b^{\prime }\in B_{\beta^{\prime }}^{j^{\prime }}$ such that there is a transition from *b* to *b′* in graph *G^B,α^*.

**Figure 1:**
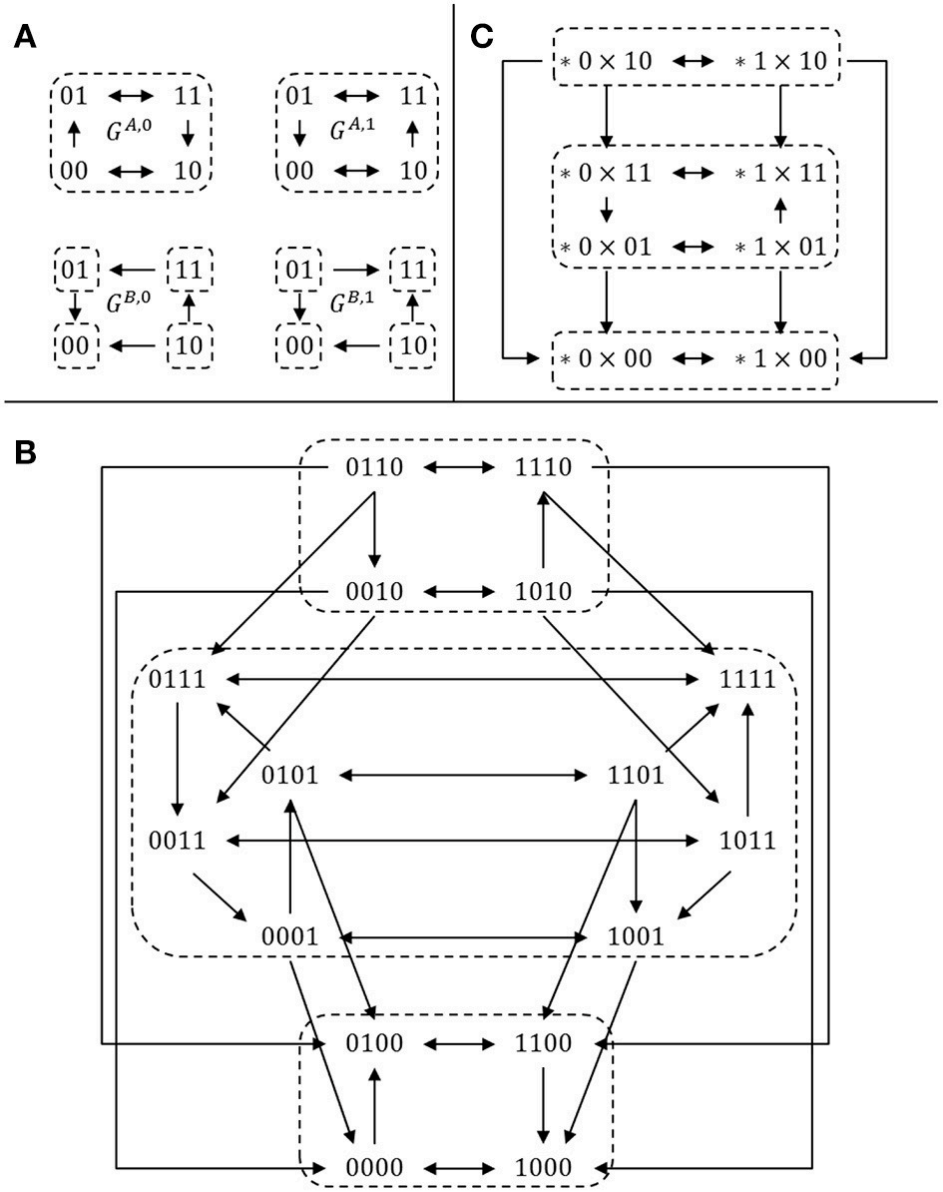
Comparison between the cross graph of an interconnection and the full transition graph. **(A)** Transition graphs of two SISO modules (see Example 4); **(B)** full transition graph *G* of the interconnection; **(C)** cross graph *G^cr^* of the interconnection. For each graph, dotted regions denote strongly connected components. There is a bijection between the SCC decomposition of the two graphs *G* (16 vertices) and *G^cr^* (8 vertices), illustrating Theorem 2.

There is also a matricial form for the definition of *G^cr^*. First, project each transition graph *G^A,u^* onto $Z_{h}^{A}$, leading to 2^*p_A_*^ graphs, represented by their *M_A_* × *M_A_* adjacency matrices *H^A,u^*, *u* ∈ {0, 1}^*p_A_*^. These projections can be rather straightforwardly achieved since $Z_{h}^{A}$ is a fragmentation of the SCC decomposition of *G^A,u^*. Second, for each α ∈ {0, 1}^*q_A_*^, introduce the *M_A_* × *M_A_* diagonal matrix $\Delta_{\alpha }^{A}$ such that entry ${\left[\Delta_{\alpha }^{A}\right]}_{ii}=1$ if the output of the *i*-th element of $Z_{h}^{A}$ is equal to α and 0 otherwise. Once similar objects *H^B,υ^* and $\Delta_{\beta }^{B}$ have been constructed for module Σ*^B^*, the cross-graph is simply defined by a generalization of formula (2):

(3) $$\begin{array}{c} G^{cr}:=\underset{(\alpha ,\beta )\in {\left\{0,1\right\}}^{q_{A}}\times {\left\{0,1\right\}}^{q_{B}}}{\vee }\left(H^{A,\beta }⊗\Delta^{B,\beta }\vee \Delta^{A,\alpha }⊗H^{B,\alpha }\right). \end{array}$$

example 4. To illustrate this definition, let us consider two 2-dimensional, single-input single-output modules Σ*^A^* and Σ*^B^*, defined by their transition graphs given in Figure 1A and their output functions *h^A^*(*x*) = *x*_2_, *h^B^*(*y*) = *y*_1_. The full transition graph of the interconnection, built from (2), is depicted in Figure 1B and the cross-graph is depicted in Figure 1C: it is constructed from the two partitions $Z_{h}^{A}=\left\{\left\{00,10\},\{01,11\right\}\right\}=\left\{\left\{*0\},\{*1\right\}\right\}$ and $Z_{h}^{B}=\left\{\left\{00\},\{10\},\{01\},\{11\right\}\right\}$.    □

The interest of the cross-graph lies in the following theorem, establishing the one-to-one correspondence between the terminal SCCs of *G^cr^* and the attractors of the interconnected network.

theorem 2. *Graphs G and G^cr^ have the same decomposition into strongly connected components. Furthermore, terminal SCCs of G^cr^ fully recover the attractors of the interconnected network*.

A proof of Theorem 2 is given in appendix. The size of the cross-graph is *M_A_* × *M_B_*, which by construction is always less or equal than 2^*n_A_*+*n_B_*^, the size of the full interconnected graph *G*. The difference in size between the two graphs may vary greatly, and strongly depends on (i) the SCC decompositions of the two modules and (ii) as for the asymptotic graph, the numbers of inputs and outputs (and therefore the general modularity of the initial network). Part 4 proposes a brief evaluation of the performance of the method for a set of randomly generated interconnections. Although the interest of the cross-graph is mainly theoretical, in certain practical cases the full graph *G* can be too big to be stored easily while *G^cr^* could.

Two possible extensions of the cross-graph method are noted here. First, Bérenguier et al. (2013) proposed a compression of the SCC graph of a network, called the hierarchical transition graph (HTG). As the cross-graph is constructed from a combination of the modules' SCC decompositions, it would be possible to consider similarly a combination of the modules' HTG decompositions. Benefiting from the compactness of HTGs, such a construction would be even more compact than the cross-graph. Second, note that both the cross graph and the asymptotic graph methods require prior analysis of the modules' dynamics and the computation of their attractors, implicitly implying the dimensions of the modules are manageable. For a large network, Zañudo and Albert (2013) proposed an efficient characterization of attractors with the notion of “stable motifs,” based on the network's interaction graph (see also Klarner et al., 2015). When considering interconnections of large modules, the investigation of the stable motifs of an interconnection would therefore constitute an interesting extension of Theorem 2.

### 3.2. A probabilistic asymptotic graph

One of the limitations of Boolean models is the lack of quantitative details: while the state transition graph describes all possible dynamical behaviors, it gives no indication as to which trajectory is more likely to be observed under a given set of initial conditions. To circumvent this problem, Boolean models can be combined with probabilistic frameworks that account for biological perturbations and variability in the logical rules (Shmulevich et al., 2002; Mori et al., 2015). Another approach is to exploit the Markov chain description of the transition graph associated to the asynchronous Boolean model (Calzone et al., 2010; Stoll et al., 2017). Based on this description, Stoll et al. (2017) developed the MaBoSS software, which then applies Gillespie algorithm to produce continuous time trajectories.

We also use the Markov chain description to assign probabilities to the edges of the asymptotic graph, an approach which will lead to a more quantitative analysis of the interconnected network's dynamics. The output of our probabilistic asymptotic graph is thus the set of attractors of the full network, under a particular interconnection scheme, together with a *relative probability* for each of them (e.g., “there is a probability *p*_1_ that phenotype *Q*_1_ is the outcome of this experiment”).

The originality of our approach consists in assigning *incidence probabilities to the attractors of each separate module*, which can be obtained through the biological observations and measurements available for each module. The goal is to include biological information as an input and provide predictions that can be confronted to biological observations and therefore lead to validate or disprove the given interconnecting scheme.

#### 3.2.1. Initializing incidence probabilities

Each transition in the asymptotic graph depends on two factors: which module is first “updated” (*A* or *B*) and, in response to an input change, how frequently does a switch occur from $A_{u\alpha }^{i}$ to $A_{\tilde{u}\tilde{\alpha }}^{k}$ (or from $B_{\upsilon \beta }^{j}$ to $B_{\tilde{\upsilon }\tilde{\beta }}^{k}$). These quantities may be represented by probabilities, defined *a priori*, from known data, experimental observations, or other modeling considerations.

Define

$$\varrho_{A}=P(\text{updating module}\;A\;\text{first}).$$

Assume Boolean module Σ*^A^* has a total of *L_A_* same-output attractor-sets and Σ*^B^* a total of *L_B_* same-output attractor-sets,

$$\{A^{\left[i\right]}:\;A_{u_{i}\alpha_{i}}^{i},\;i=1,\ldots ,L_{A}\},\;\;\{B^{\left[j\right]}:\;B_{\upsilon_{j}\beta_{j}}^{j},\;j=1,\ldots ,L_{B}\},$$

and each of these has a given *incidence probability* (meaning that it is observed with a certain frequency) defined as

$$P(A^{\left[i\right]})=w_{A}^{i},\;\;i=1\ldots L_{A},\;\;\;\;P(B^{\left[j\right]})=w_{B}^{j},\;\;\;\;j=1\ldots L_{B}.$$

The probabilities $w_{A}^{i}$ and $w_{B}^{j}$ may be assigned in different ways, for instance using experimental observations, or setting uniform probabilities ($w_{A}^{i}=1/L_{A}$ for all *i*), or else from the size of their respective basin of attraction

(4) $$\begin{array}{c} w_{A}^{i}=\frac{\#{\text{basin}}^{i}}{\sum_{i}\#{\text{basin}}^{i}}, \end{array}$$

but in any case they should satisfy $\sum_{i=1}^{L_{A}}w_{A}^{i}=1.$ Using these initial probabilities, a *joint incidence probability* may similarly be defined for each product of attractor-sets:

$$P(A^{\left[i\right]}\times B^{\left[j\right]})=w_{A}^{i}w_{B}^{j},\;\;\;\;\Rightarrow \;\;\sum_{i=1}^{L_{A}}\sum_{j=1}^{L_{B}}P(A^{\left[i\right]}\times B^{\left[j\right]})=1.$$

#### 3.2.2. Transition probabilities in the asymptotic graph

The probability of switching between two attractor-sets of the same module, but different inputs, can be defined in terms of conditional probabilities: define $s_{A}^{ik}$ to be the probability that attractor *A*^[*k*]^ is reached, conditional to the fact that the initial state is some *a^i^* ∈ *A*^[*i*]^. In other words, $w_{A}^{k}$ must be weighted by the probability of *a^i^* reaching any attractor in *G^A,u_k_^*:

(5) $$\begin{array}{c} s_{A}^{ik}=P(A^{\left[k\right]}|[a^{i}\in A^{\left[i\right]}])=\frac{P(A^{\left[k\right]})}{\sum_{j\in J}P(A^{\left[j\right]})}=\frac{w_{A}^{k}}{\sum_{j\in J}w_{A}^{j}}, \end{array}$$

where J = {*j* : *u_j_* = *u_k_* and *a^i^* ⇝ *A*^[*j*]^} means that there exists a path in *G^A,u_k_^* leading from *a^i^* to *A*^[*j*]^, where *A*^[*j*]^ is an attractor of *G^A,u_k_^*. A similar definition holds for $s_{B}^{ik}$.

Next, we can define the probability associated to an edge of *V^as^* as:

(6) $$\begin{array}{l} P(A^{\left[i\right]}\times B^{\left[j\right]}\to A^{\left[k\right]}\times B^{\left[j\right]})\;=\;{\bar{\varrho }}_{A}s_{A}^{ik},\\ P(A^{\left[i\right]}\times B^{\left[j\right]}\to A^{\left[i\right]}\times B^{\left[k\right]})\;=\;(1-{\bar{\varrho }}_{A})s_{B}^{jk}, \end{array}$$

with an “effective” probability ${\bar{\varrho }}_{A}$, computed based on the set of all ougoing edges from node *A*^[*i*]^ × *B*^[*j*]^:

(7) $$\begin{array}{c} {\bar{\varrho }}_{A}=\{\begin{array}{ll} 0, & A^{\left[i\right]}\equiv A^{\left[k\right]}\\ 1, & B^{\left[j\right]}\equiv B^{\left[k\right]}\\ \varrho_{A}, & \text{otherwise}. \end{array} \end{array}$$

In other words, ${\bar{\varrho }}_{A}=0$ if all outgoing edges have a fixed *A*-attractor, *A*^[*i*]^ × *B*^[*j*]^ → *A*^[*i*]^ × *B*^[*k*]^; ${\bar{\varrho }}_{A}=1$, if all outgoing edges have a fixed *B*-attractor *A*^[*i*]^ × *B*^[*j*]^ → *A*^[*k*]^ × *B*^[*j*]^; ${\bar{\varrho }}_{A}=\varrho_{A}$ if outgoing edges may be of both types.

Note that these definitions ensure that the probabilistic asymptotic graph matrix has the property that all rows add up to 1:

$$\begin{array}{l} \sum_{k}P(A^{\left[i\right]}\times B^{\left[j\right]}\to A^{\left[k\right]}\times B^{\left[j\right]})\\ \;+\sum_{k}P(A^{\left[i\right]}\times B^{\left[j\right]}\to A^{\left[i\right]}\times B^{\left[k\right]})\\ \;=\sum_{k}{\bar{\varrho }}_{A}s_{A}^{ik}+\sum_{k}\left(1-{\bar{\varrho }}_{A}\right)s_{B}^{jk}={\bar{\varrho }}_{A}+(1-{\bar{\varrho }}_{A})=1 \end{array}$$

since both $\sum_{k}s_{A}^{ik}=1$ and $\sum_{k}s_{B}^{jk}=1$.

#### 3.2.3. Relative probabilities of the attractors of an interconnection

If the asymptotic graph *G^as^* has two or more attractors, in addition to the transition probabilities, another useful information is the frequency of observing a given attractor, or in other words the *relative probability of each attractor* of the interconnection. This probability can be computed from the SCC graph *G^Sd^* = (*V^Sd^*, *E^Sd^*) corresponding to *G^as^*, which is an acyclic graph and can be represented by an absorbing Markov chain. By definition, *V^Sd^* is composed of the strongly connected components of *G^as^*. Let *C* ∈ *V^Sd^* contain *L_C_* elements of *V^as^*. Define the incidence probability of observing *C* as:

$$P(C)=\sum_{\ell =1}^{L_{C}}P(A^{\left[i(\ell )\right]}\times B^{\left[j(\ell )\right]})=\sum_{\ell =1}^{L_{C}}w_{A}^{i(\ell )}w_{B}^{j(\ell )}.$$

Moreover, a probability of transition can also be associated to each edge of *E^Sd^*, *P*(*C^i^* → *C^j^*), computed by adding all the probabilities of the edges in *E^as^* that link elements of *C^i^* to elements of *C^j^*. Suppose there are *m* strongly connected components, |*V^Sd^*| = *m*, and let the *m* × *m* matrix *M* with *M_ij_* = *P*(*C^i^* → *C^j^*), be the absorbing Markov chain associated with the graph *G^Sd^*. Suppose *M* has *r* absorbing states, $\left\{C_{a}^{k}:k=1,\ldots ,r\right\}$, these are also the attractors of *G^Sd^*. Matrix *M* can be written in the following canonical form (Feller, 1970):

$$M=\left[\begin{array}{cc} Q & R\\ 0 & I_{r} \end{array}\right],$$

where *I_r_* is the *r*×*r* identity matrix, *Q* is the (*m* − *r*) × (*m* − *r*) matrix of transitions between transient states and *R* is the (*m* − *r*) × *r* matrix of transitions from transient states to absorbing states. Since *M* is irreducible, it follows that (*I*−*Q*) has an inverse (where *I* is the (*m*−*r*) × (*m*−*r*) identity matrix). Then the probability that there exists a path from a given state to one of the *r* absorbing states is given by the probability of being absorbed by *r*:

$$M_{absorp}={\left(I-Q\right)}^{-1}R,$$

where *M_absorp_*(*i, k*) is the probability that transient state *i* converges to absorbing state *k*.

If, in addition, we wish to weigh these absorption probabilities by the incidence probabilities of observing $C_{a}^{k}$, we can define the *relative probability of an attractor of the asymptotic graph*:

(8) $$\begin{array}{c} P_{rel}(C_{a}^{k})=P(C_{a}^{k})+\sum_{i=1}^{m-r}M_{absorp}(i,k)P(C^{i}),\;\;\;\;k=1,\ldots ,r \end{array}$$

where $C_{a}^{k}$ denotes each attractor and $P\left(C_{a}^{k}\right)$ is the incidence probability of $C_{a}^{k}$.

## 4. Performance on random networks' interconnections

In this part we propose a series of computational experiments to assess the efficiency of the asymptotic graph and the cross graph to recover the attractors of random interconnected Boolean networks. Following the general idea of inputs/outputs at the core of this paper, we start with a brief description of the algorithm used to generate random IO modules. We then present numerical results computed on random interconnections with varying connectivity, showing the respective advantages and limitations of the two methods in practice.

### 4.1. Generation of random io networks with varying connectivity

The NK-model, introduced by Kauffman (1969), is a general statistical model to represent random Boolean networks by controlling their dimension *N* and their inner connectivity *K*. It is used for instance by Zañudo and Albert (2013) and Veliz-Cuba et al. (2014). Here it is slightly adapted to include inputs and outputs. Let Σ be an IO Boolean network of dimension (*n, p, q*), of transition function *f* : {0, 1}^*p*^ × {0, 1}^*n*^ → {0, 1}^*n*^ and output function *h*:{0, 1}^*n*^ → {0, 1}^*q*^. A usual way to depict such a network is by its wiring diagram, showing the dependencies between the different variables of the network. Equivalently, the wiring diagram can be represented by a (*n* + *q*) × (*p* + *n*) Boolean matrix 
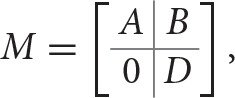
 where submatrices *A* (*n* × *p*), *B* (*n* × *n*) and *D* (*q* × *n*) are defined as follows:

$$\begin{array}{l} a_{ij}=\{\begin{array}{l} 1\text{ if function }f_{i}\text{ depends explicitly of input variable }u_{j},\\ 0\text{ otherwise,} \end{array}\\ b_{ij}=\{\begin{array}{l} 1\text{ if function }f_{i}\text{ depends explicitly of variable }x_{j},\\ 0\text{ otherwise,} \end{array}\\ d_{ij}=\{\begin{array}{l} 1\text{ if output function }h_{i}\text{ depends explicitly of variable }x_{j},\\ 0\text{ otherwise}\text{.} \end{array} \end{array}$$

Let *C* designate the matrix [*A*|*B*]. The sum of the *i*-th row of *C* is the number of essential variables of logical function *f_i_*, also called the *connectivity* of *f_i_*. Given integers *n* > 0, *p, q* ≥ 0 and a real number *K_mean_* ∈ [1, *n*], we construct a random IO network of dimension (*n, p, q*) and of average connectivity *K_mean_* by applying the following procedure, which generates a dependency matrix *M*:

1. Let *D*: = 0. For each 1 ≤ *i* ≤ *q*, pick at random *j* ∈ {1, …, *n*} and set *d_ij_*: = 1.
2. Generate *n* integers *k_i_* in {0, …, *n*+*p*} according to a binomial distribution of parameters *n* + *p* (number of trials) and $\frac{K_{mean}}{n+p}$ (probability of success).
3. Let *C* = [*A*|*B*]: = 0. For each 1 ≤ *i* ≤ *n*, pick a random combination (*j*_1_, …, *j_k_i__*) ∈ {1, …, *n* + *p*}^*k_i_*^ (without replacement) and set *c_i,j_l__*: = 1 for all 1 ≤ *l* ≤ *k_i_*.
4. Check that each column of *A* is non-zero; while it is not the case, repeat step 3.
5. Set $M:=\left[\frac{C}{0|D}\right]$.

Step 4 ensures the generated module actually depends of every inputs. Once the dependency matrix *M* is obtained, the last step consists in generating the *n*+*q* Boolean functions according to *M*. A Boolean function of *k* variables is picked randomly among the 2^2^*k*^^ possibilities; in case it is degenerate (i.e., at least one of the *k* variables is not essential), another one is chosen so as to ensure exact compatibility with *M*.

### 4.2. Complementarity of the cross and asymptotic graph methods

**Figure 2:**
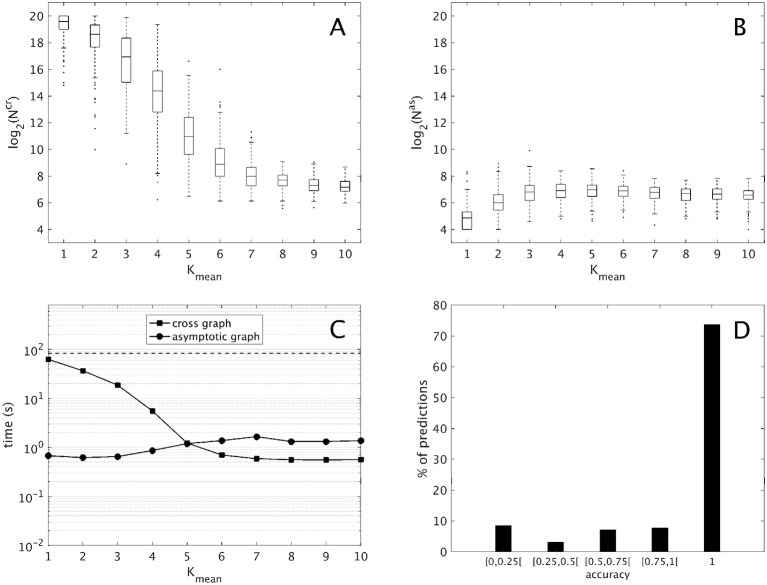
Computational results for 2,000 interconnections of two 10-dimensional modules [according to (9)], split into ten groups of 200 sorted along *K_mean_*, the mean connectivity of the modules. **(A,B)** Evolution of the sizes of the cross graph and of the asymptotic graph, log_2_(*N^cr^*) and log_2_(*N^as^*) with respect to the modules' connectivity (obtained with the routine boxplot of Matlab's Statistics toolbox). **(C)** Mean execution time in seconds of the cross and asymptotic graph methods (logarithmic scale). The dotted line represents the average time of the direct method (analysis of the full interconnected network). **(D)** Histogram of the accuracies of all the attractors predicted by the asymptotic graph (3,693 attractors in total).

With this algorithm, it is possible to generate a IO module by controlling its inner connectivity, that is the number of actual dependencies in the wiring diagram. Thus, it becomes possible to generate random interconnections with varying degrees of *modularity*, according to the average connectivity of each module. We used this algorithm to generate 2,000 interconnections of two modules Σ*^A^* and Σ*^B^* of dimensions (*n_A_*, *p_A_*, *q_A_*) = (*n_B_*, *p_B_*, *q_B_*) = (10, 2, 2): 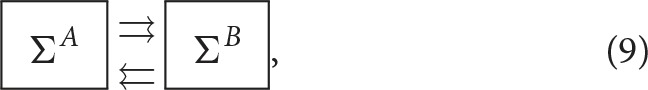


where the mean connectivity of Σ*^A^* and Σ*^B^* varies in {1, …, 10}. For each interconnection, both 10-dimensional modules were analyzed separately (including the computation of the transition graphs, their SCC decompositions and the computation of their attractors), then the cross graph and the asymptotic graph were computed and compared. The main results are presented in Figure 2 and summarized below. All computations were made with Matlab R2016b, The MathWorks, Inc.

First, we compare the respective sizes *N^cr^* and *N^as^* of the cross and the asymptotic graphs (*ie*. their number of vertices). Figures 2A,B show respectively the evolution of log_2_(*N^cr^*) and log_2_(*N^as^*) with respect to the connectivity of the two modules. Obviously, both *N^cr^* and *N^as^* are below *N* = 2^20^, which is the size of the full transition graph of the interconnected network. The cross graph, which captures both the transient and the asymptotic dynamics of the interconnection is relatively large, however its size seems to vary greatly with the modules' connectivity. When the connectivity increases, implying a highly modular interconnection, the ratio *N^cr^*/*N* can reach very small values, emphasizing the interest of the cross graph to efficiently store the dynamics of large, modular interconnected networks. On the other hand, the asymptotic graph is always much smaller, several orders of magnitude under the size of the full transition graph. Contrary to the cross graph, it is particularly small when the modules have lower connectivity, making it particularly well adapted for biological networks. Interestingly, its size seems to reach a plateau when the mean connectivity is above $\frac{n}{2}=5$.

Another way to compare the two approaches is by studying their average execution times. The times shown in Figure 2C include the analysis of the two 10-d modules and of the cross and asymptotic graph methods. The latter comprise the construction of *G^cr^* (respectively, of *G^as^*), the SCC decomposition of *G^cr^* (respectively, of *G^as^*) and the reconstruction of the attractors (respectively, of π(*R*) for all terminal SCCs *R* of *G^as^*). For the cross graph, the majority of the time is taken by the SCC decomposition of *G^cr^* while for the asymptotic graph, the most time-consuming step is the construction of *G^as^* itself (data not shown). For comparison, we also computed the complete dynamics of the 20-d interconnected network by using formula (2); on average, such direct method amounted to around 83 seconds (dotted line). Therefore, both methods are faster than the direct analysis of the full interconnected network. As before, the asymptotic graph is particularly efficient for low connectivity modules, while the cross graph is more efficient when the modules have high connectivity. Interestingly, for connectivity *K_mean_* = 5 and higher, when both graphs have roughly the same size, the cross graph method becomes even more rapid than the asymptotic graph.

Finally, since both graphs were computed it was possible to evaluate the quality of the asymptotic graph predictions. Recall that according to Theorem 1, the asymptotic graph has two drawbacks. First, it may predict spurious attractors and second, when it identifies a true attractor it only predicts a subset π(*R*) of the states lying in the attractor *Q*. The ratio $\frac{\left|\pi \left(R\right)\right|}{\left|Q\right|}$ is called the *accuracy* of the prediction. Among the 2,000 interconnections, 11 presented spurious attractors that is only 0.55% of the total. In all but one case, only one spurious attractor was detected. This result confirms the rarity of the appearance of spurious attractors. In total, we identified 3,693 true attractors. Among them more than 73% were completely recovered (see Figure 2D); overall, the mean accuracy is about 0.86, exhibiting the excellent predictive power of the asymptotic graph when it comes to uncover the asymptotic behaviors of an interconnection.

### 4.3. A powerful tool to analyze large interconnections of biological networks

According to the previous results, the asymptotic graph seems particularly well adapted when the mean connectivity of the modules is low (≤ 5), which is arguably where biological networks generally operate (Zañudo and Albert, 2013; Veliz-Cuba et al., 2014). Therefore we decided to test it further with higher dimensional interconnections, including four modules Σ*^A^*, Σ*^B^*, Σ*^C^*, Σ*^D^* of dimension *n* = 15, with *K_mean_* ∈ {1, …, 5}, *p_A_* = *q_A_* = *p_D_* = *q_D_* = 1 and *p_B_* = *q_B_* = *p_C_* = *q_C_* = 2: 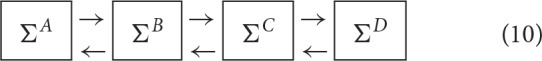


When *N^cr^* < 10^7^, the cross graph was also constructed and analyzed, in order to check the existence of spurious attractors. Since the global state space is 2^60^ > 10^18^, we skipped the last treatment (identification of the attractors in {0, 1}^60^) to avoid possible explosions. Therefore, we only computed the terminal SCCs of *G^as^* and, when available, the terminal SCCs of *G^cr^*. The results are presented in Table 1. When *G^cr^* could be analyzed, we were able to detect spurious attractors in *G^as^*: none were found. If the cross graph method is not practical for small *K_mean_*, the asymptotic graph was always manageable, confirming its practical interest to analyze large biological networks, as long as they can be expressed as interconnections of modules with a reasonable number of inputs and outputs.

**Table 1:** Computational results for 200 interconnections of four 15-dimensional modules [according to (10)], split into five groups of 40, sorted along the mean connectivity of the modules.

***K_mean_***	**log_2_**(***N^cr^***)		**Time (*****s*****)**			**log_2_**(***N^as^***)		**Time (*****s*****)**		
	**mean**	**std**	**mean**	**std**	**#treated/(#exp.)**	**mean**	**std**	**mean**	**std**	**#spurious/(#treated)**
1	57.3	2.3	−	−	0/40	8.5	1.4	9	2	−
2	52.2	4.1	−	−	0/40	9.8	1.1	9	7	−
3	42.4	5.7	−	−	0/40	11.0	1.4	63	185	−
4	29.6	5.9	493	361	6/40	11.3	1.1	40	51	0/6
5	20.9	4.7	176	223	28/40	11.0	1.0	27	38	0/28

*The cross graph is treated (constructed and analyzed) only when N^cr^ < 10^7^ (log_2_(10^7^) ≈ 23.25). The column #treated/(#exp.) indicates the number of times it was treated over the total number of experiments. When it is treated, we further verify the presence of spurious attractors in the asymptotic graph. The column #spurious/(#treated) indicates the number of times the asymptotic graph predicts a spurious attractor over the number of times the cross graph could be treated. Symbol — indicates that the corresponding value could not be computed*.

## 5. Two biological applications

The asymptotic graph construction and its probabilistic interpretation are now applied to two biological examples, centered on the mammalian and yeast cell cycles. Both cases illustrate the asymptotic graph concept, its informative description of a composite system, and its usefulness for testing biological hypotheses.

### 5.1. Mammalian cell cycle, circadian clock and their interconnection

There are two basic cellular oscillators in mammalian cells: cell cycle describes the different phases of cellular growth and division, while circadian clock decribes the mechanism responsible for anticipating environmental changes and adapting the organism to deal with these changes (most notably, day-night differences). The interactions between these two oscillators are still not fully understood, but recent works by Feillet et al. (2014) and Bieler et al. (2014) have uncovered unexpected bi-directional links between the two modules. Successful mathematical models for the cell cycle and clock have been developed, as well as some studies on their interactions (Gérard and Goldbeter, 2012), but many questions remain (Feillet et al., 2015).

#### 5.1.1. Mammalian boolean modules

**Figure 3:**
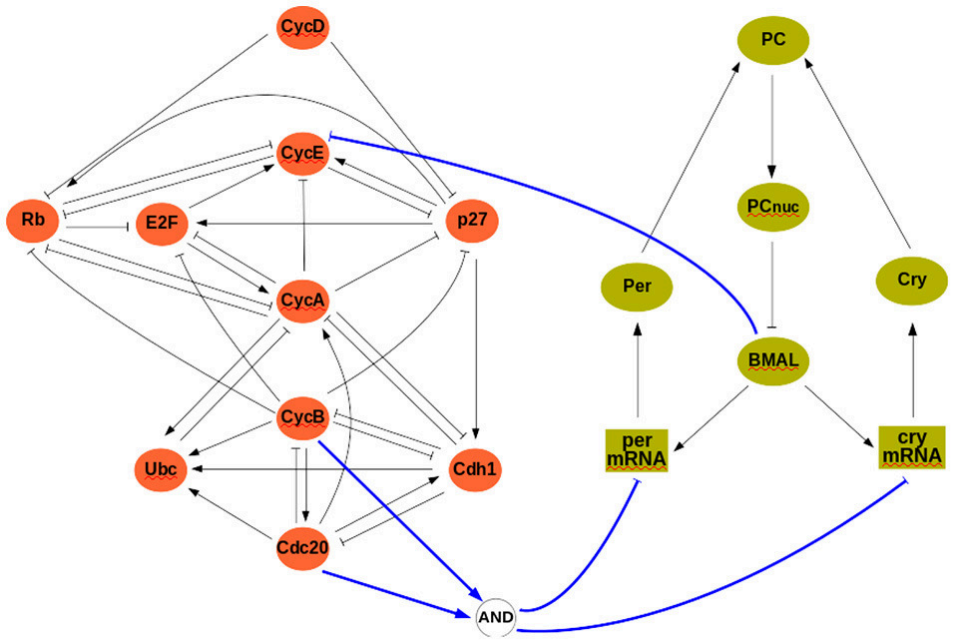
The interconnected mammalian cell cycle (**Left**, adapted from Fauré et al., 2006) and clock (**Right**, adapted from Comet et al., 2012). Square symbols represent messenger RNAs. Solid blue arrows denote input/output connections.

At the discrete level, a reference model of the cell cycle was developed and discussed by Fauré et al. (2006) (see Figure 3). It comprises 10 variables:

(CycD,Rb,E2F,CycE,CycA,p27,Cdc20,Cdh1,Ubc,CycB),

where *CycX* (*X* ∈ {*A, B, D, E*}) represent four cyclins, each roughly corresponding to one of the four phases of the cell cycle. This constitutes our module Σ*^A^*, and its rules can be found in the Supplementary Material. The clock model (module Σ*^B^*) has 7 variables and is based on the work of Comet et al. (2012). To account for transcription shutdown during mitosis, the input *v* negatively affects all mRNAs:

(11) $$\begin{array}{l} \;BMAL^{+}=\neg PCnuc\\ \;mPER^{+}=\neg \upsilon \wedge BMAL\\ \;mCRY^{+}=\neg \upsilon \wedge BMAL\\ \;\;pPER^{+}=mPER\\ \;\;pCRY^{+}=mCRY\\ \;PC^{+}=pPER\wedge pCRY\\ PCnuc^{+}=PC. \end{array}$$

In the clock model, *mX* and *pX* denote mRNA and protein coded by gene *X*, while *PC* denotes the complex formed by the proteins PER and CRY, and *PCnuc* denotes this complex in the nucleus.

A well established link between these two oscillators is that protein *BMAL* acts on the cell cycle, possibly at different stages (Feillet et al., 2015). In our analysis, we will consider *BMAL* acting during G1 phase. Although no conclusive evidence exists on how the cell cycle may affect the clock, we have considered that during cell division (or mitosis phase) gene expression is stopped (in the model, mitosis can be modeled as *Cdc*20∧*CycB*, see Figure 3). The interconnection between modules is thus given by:

$$u=h^{B}(b)=BMAL,\;\;\;\;\;\;\upsilon =h^{A}(a)=Cdc20\wedge CycB,$$

so that *u* = 0 (resp., *u* = 1) represents absence (resp., presence) of *BMAL* and υ = 1 represents mitosis. In the cell cycle model, *BMAL* affects negatively the G1 phase, leading to a logical equation for cyclin E of the form *cycE*^+^ = ¬*u*∧(*E*2*F*∧¬*Rb*) (see Figure 3 and Supplementary Material).

Module Σ*^A^* has a total of six, and module Σ*^B^* has a total of three, same-output attractor sets. For algorithmic convenience, these are labeled using the lexicographic convention, that is $A_{\hat{u}\hat{\alpha }}^{j}$ for $û,\hat{\alpha }\in \left\{1,2\right\}$, where “decimal 1 = logical 0” and “decimal 2 = logical 1.” The attractors for both modules are as follows:

$$\begin{array}{l} G^{A,u=0}:\;A_{11}^{1}=\{0100010100\},\;A_{11}^{2}(\text{80 states}),\;A_{12}^{3}(\text{32 states}),\\ G^{A,u=1}:\;A_{21}^{4}=\{0100010100\},\;A_{21}^{5}(\text{40 states}),\;A_{22}^{6}(\text{16 states}),\\ G^{B,v=0}:\;B_{11}^{1}(\text{57 states}),\;B_{12}^{2}(\text{63 states}),\\ G^{B,v=1}:\;B_{22}^{3}=\{1000000\}. \end{array}$$

In the case *u* = 0, module Σ*^A^* becomes exactly the original model constructed by Fauré et al. (2006). Therefore, as expected, the attractors found for *G*^*A,u*=0^ correspond exactly to those listed by Fauré et al. (2006). Attractors $A_{11}^{1}$ and $A_{21}^{4}$ correspond to a steady state where the only expressed proteins are *Rb*, *p*27, and *Cdh*1, hence representing the quiescent cell state. The (full) attractor $A_{11}^{2}\cup A_{12}^{3}$ is a cyclic attractor containing 112 distinct states and corresponds to the known G1/S/G2/M cell cycle progression (Fauré et al., 2006). Similarly, $A_{21}^{5}\cup A_{22}^{6}$ is a cyclic attractor of the graph *G*^*A,u*=1^, with 56 states. It tends to describe the cell cycle progression, with the difference that *u* = 1 implies *CycE* ≡ 0. In either of the cyclic attractors, the attractor-sets $A_{12}^{3}$ and $A_{22}^{6}$ contain states representing mitosis, that is, the output of any state $a\in A_{22}^{6}\cup A_{12}^{3}$ satisfies *h^A^*(*a*) = *Cdc*20 ∧ *CycB* = 1.

The clock mechanism admits a cyclic attractor with 120 states, $B_{11}^{1}\cup B_{12}^{2}$, which corresponds to regular circadian oscillations in the case υ = 0. At mitosis, represented by υ = 1, the clock network admits a single steady state attractor ($B_{22}^{3}=\{1000000\}$), where all gene expression is arrested.

#### 5.1.2. Asymptotic and cross graphs

**Figure 4:**
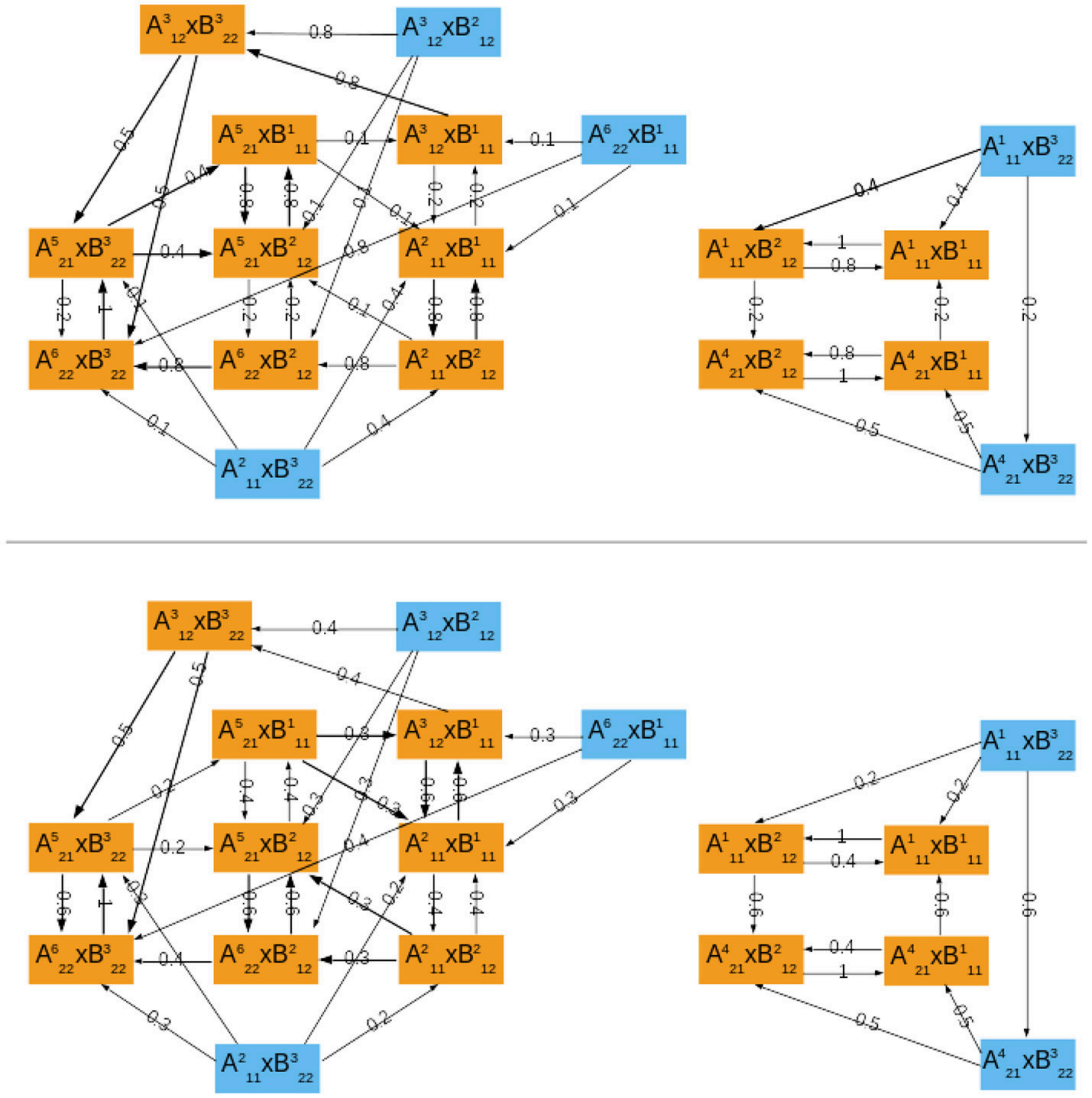
The probabilistic asymptotic graph for the interconnected mammalian oscillators. Orange colored nodes belong to an attractor: *R*_1_ at right and *R*_2_ at left. Bold arrows represent transitions with probability ≥ 0.5. **(Top)** ϱ*_A_* = 0.2. **(Bottom)** ϱ*_A_* = 0.6.

The asymptotic graph for the interconnection of the two mammalian oscillators has 18 nodes and two attractors, with separate basins of attraction (Figure 4):

$$\begin{array}{l} R_{1}\;=\{A_{11}^{1}\times (B_{11}^{1}\cup B_{12}^{2}),A_{21}^{4}\times (B_{11}^{1}\cup B_{12}^{2})\}\\ R_{2}\;=\{(A_{11}^{2}\cup A_{12}^{3})\times B_{11}^{1},A_{11}^{2}\times B_{12}^{2},A_{21}^{5}\times (B_{11}^{1}\cup B_{12}^{2}),\\ \;\;\;\;\;\;\;\;\;\;\;(A_{21}^{5}\cup A_{22}^{6})\times B_{22}^{3},A_{22}^{6}\times B_{12}^{2},A_{12}^{3}\times B_{22}^{3}\}. \end{array}$$

The cross graph contains 54,272 nodes (compare to the full size of the interconnection, 2^17^ = 131072) and confirms the existence of exactly two cyclic attractors for the interconnected system and returns all their elements: attractor *R*_1_ is composed of 120 states and *R*_2_ is composed of 13,552 states.

Our methodology predicts two distinct operating modes for the coupled oscillators: *R*_1_ corresponds to a quiescent cell with oscillatory clock, since it is the product of state 0100010100 representing a quiescent cell in module Σ*^A^* and of cyclic attractor $B_{11}^{1}\cup B_{12}^{2}$ representing regular clock oscillations. The attractor *R*_1_ is thus in agreement with observations by Plikus et al. (2013) (hair cells in quiescent phase seem to have a running clock). In contrast, *R*_2_ represents joint oscillations of the cell progression cycle ($A_{11}^{2}\cup A_{12}^{3}$) and clock ($B_{11}^{1}\cup B_{12}^{2}$) (see Figure 4 for the dynamics within *R*_2_). The cell cycle and clock may jointly oscillate and alternate states with a regular cycle of cyclin E (which is present mostly through S phase and mitosis) or eventually switch to a joint cycle with absence of cyclin E ($A_{21}^{5}\times B_{1\cdot }^{1}\to A_{1\cdot }^{2}\times B_{11}^{1}\to A_{11}^{2}\times B_{12}^{2}\to A_{21}^{5}\times B_{1\cdot }^{1}$). However, at mitosis ($A_{12}^{3}$), the clock may switch to its arrested steady state ($A_{12}^{3}\times B_{11}^{1}\to A_{12}^{3}\times B_{22}^{3}$), which leads directly to a full degradation of cyclin E in the cell cycle ($A_{2\cdot }^{5}\times B_{22}^{3}$).

To assign transition probabilities to the asymptotic graph, there are essentially two elements to define: ϱ*_A_* which is the probability of updating first the component from module Σ*^A^*; and the incidence probability of each attractor from each module, $w_{A}^{i}$ and $w_{B}^{j}$. To compute the incidence probabilities $w_{A}^{i}$ and $w_{B}^{j}$, we have used the size of the original basins of attraction of $A_{u\alpha }^{i}$ in Σ*^A^* and $B_{\upsilon \beta }^{j}$ in Σ*^B^*, as in (4). However, for both modules, each attractor can be reached from any state, implying that the joint incidence probabilities, $P(A^{\left[i\right]}\times B^{\left[j\right]})=w_{A}^{i}\times w_{B}^{i}$, are equal for all nodes of the asymptotic graph with: $w_{A}^{i}=1/6$ (*i* = 1, …6) and $w_{B}^{j}=1/3$ (*j* = 1, …3).

Figure 4 shows the transition probabilities obtained for two different values of the updating probability ϱ*_A_*. These two graphs are very similar, differing only on the most frequent transitions (bold arrows, above 0.5). As should be expected, whenever the probability of first updating components from Σ*^A^* is larger (ϱ*_A_* = 0.6), the cell cycle oscillations dominate the global dynamics: most of the bold transitions in Figure 4 (bottom) concern switches between attractor-sets of Σ*^A^*. In contrast, circadian clock oscillations are dominant for ϱ*_A_* = 0.2 (Figure 4, top). The evolution from mitosis phase toward cell cycle progression ($A_{12}^{3}\times B_{22}^{3}\to A_{21}^{5}\times B_{22}^{3}\text{ or }A_{12}^{3}\times B_{22}^{3}\to A_{22}^{6}\times B_{22}^{3}$) is equally probable for either ϱ*_A_*.

Computation of the relative probabilities (8) of reaching one of the attractors of the interconnected network yields

$$P_{rel}(R_{1})=0.333,\;\;\;P_{rel}(R_{2})=0.667,$$

independently of the updating probability ϱ*_A_*. An interpretation of these relative probabilities is that, in a typical population of cells, about one third are arrested in quiescent G0 state while the other two thirds follow the normal cell cycle progression G1/S/G2/M.

### 5.2. Budding yeast cell growth and cell cycle start

Cell cycle and division is intimately linked with cell growth: a cell cannot divide into two daugther cells if its size is too small. There are many other factors that play a role in cell division (concentration of certain proteins, volume), but it remains unclear how a cell is able to perceive its own size and evaluate whether all conditions are in place for cell division (Turner et al., 2012).

In budding yeast, cell cycle is triggered by a START signal which is dependent on cell size. Li et al. (2004) propose a Boolean model that accurately describes cell cycle progression, taking START as an external input and stopping at a G1 phase steady state. One of the most important proteins involved in START is cyclin *Cln*3, which in involved in the G1-S phase transition and initiates cell cycle in the model of Li et al. (2004). Cyclin *Cln*3 forms a complex with another protein *Whi*3 but, in order to initiate cell cycle, *Cln*3 must be folded and released from this complex, which is achieved with the help of a chaperon protein *Ydj*1. Recent work by Aldea et al. (2017) suggests that cell size is growth rate dependent and that *Ydj*1 is one of the most important factors relating growth rate to cell size at START.

#### 5.2.1. Budding yeast boolean modules

**Figure 5:**
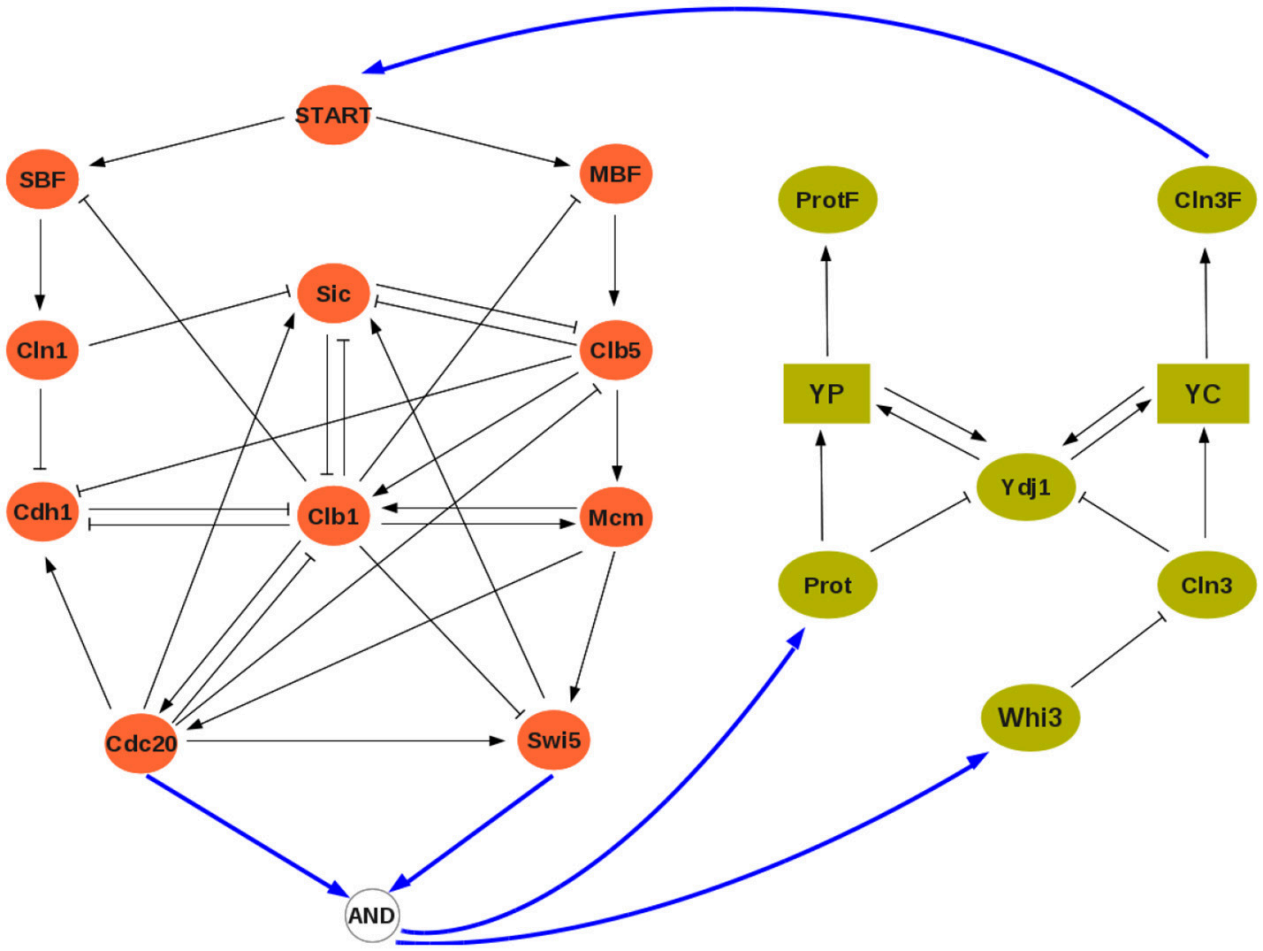
The interconnected yeast cell cycle (**Left**, adapted from Li et al., 2004) and cell size network (**Right**, adapted from Aldea et al., 2017). Square symbols represent messenger RNAs. Solid blue arrows denote input/output connections.

A reference discrete model for the cell cycle was developed by Li et al. (2004). It comprises 11 variables:

$$(START,MBF,SBF,Cln1,Cdh1,Swi5,Cdc20,Clb5,Sic1,Clb1,Mcm{)}^{\prime }$$

with *START* given by *Cln*3 (see Figure 5; the Boolean rules can be found in the Supplementary Material).

To describe cell size dependence on growth rate Aldea et al. (2017) proposes a model where *Cln*3 competes with a second hypothetical protein *Prot* for binding with *Ydj*1 for folding:

$$\begin{array}{l} Prot+Ydj1⇋YP\to ProtF+Ydj1\\ Cln3+Ydj1⇋YC\to Cln3F+Ydj1, \end{array}$$

and *Prot* would be a growth rate dependent protein. Here, we propose a basic Boolean network of this model, where the dependence on growth rate is modeled by an input υ:

(12) $$\begin{array}{l} \;\;\;\;\;Ydj1^{+}\;=YP\vee YC\vee \neg (Prot\wedge Cln3)\\ \;\;\;\;\;\;\;\;YP^{+}\;=Ydj1\wedge Prot\\ \;\;\;\;\;\;\;\;YC^{+}\;=Ydj1\wedge Cln3\\ \;\;\;\;Prot^{+}\;=\upsilon \\ \;ProtF^{+}\;=YP\\ \;\;\;\;Cln3^{+}\;=\neg Whi3\\ \;\;Cln3F^{+}\;=YC\\ \;\;\;Whi3^{+}\;=\upsilon . \end{array}$$

The competition of *Prot* and *Cln*3 for *Ydj*1 is represented by the term ¬(*Prot* ∧ *Cln*3) in the rule for *Ydj*1 meaning that, in the absence of both *Prot* and *Cln*3, “free” protein *Ydj*1 will be available. Both *Prot* and *Whi*3 depend on growth rate, here given by input υ. Later on, υ will be computed as an output from the cell cycle model.

Computation of the graphs *G^A,u^* and *G^B,v^* yields the following attractors:

$$\begin{array}{l} G^{A,u=0}:\;A_{11}^{1}=\{00000000000\},\;A_{11}^{2}=\{00000000100\},\\ \;\;\;\;\;\;\;\;\;\;\;\;\;\;\;\;\;A_{11}^{3}=\{00001000000\},\\ \;\;\;\;\;\;\;\;\;\;\;\;\;\;\;\;\;A_{1*}^{4}=\{00001000100\},\;A_{11}^{5}=\{00110000000\},\\ \;\;\;\;\;\;\;\;\;\;\;\;\;\;\;\;\;A_{11}^{6}=\{01000000100\},\\ \;\;\;\;\;\;\;\;\;\;\;\;\;\;\;\;\;A_{1*}^{7}=\{01001000100\},\\ G^{A,u=1}:\;A_{2*}^{8}=\{10110110011\},\;A_{2*}^{9}=\{11000111011\},\\ \;\;\;\;\;\;\;\;\;\;\;\;\;\;\;\;\;A_{2*}^{10}=\{11110110011\},\\ \;\;\;\;\;\;\;\;\;\;\;\;\;\;\;\;\;A_{2*}^{11}=\{11110111011\},\\ G^{B,\upsilon =0}:\;B_{12}^{1}=\{10100110\},\\ G^{B,\upsilon =1}:\;B_{21}^{2}=\{11011001\}. \end{array}$$

The symbol * in $A_{1*}^{i}$ or $A_{2*}^{i}$ means that the output of this attractor depends on the function *h^A^*(*a*): three different forms for *h^A^*(*a*) will be tested (see 13–15 below). For instance, we have $h^{A}(A_{1*}^{4})=2$ whenever *h^A^*(*a*) is given by (15), so we should write $A_{12}^{4}$; but $h^{A}(A_{1*}^{4})=1$ in the other two cases, hence $A_{11}^{4}$.

In the case *u* = 0, the yeast cell cycle model is exactly the one studied by Li et al. (2004) hence, as expected, the seven attractors $A_{1*}^{i}$ of *G*^*A,u*=0^ are those listed in Table 1 of this reference. According to Li et al. (2004), attractor $A_{1*}^{4}$ represents the G1 steady state and has the largest attraction basin. Attractor $A_{11}^{2}$ is also close to G1 phase and has the second largest attraction basin. Using the size of the attractions basins, the incidence probabilities $w_{A}^{i}$ have been computed according to Equation (4) and they are listed in Table 2.

**Table 2:** Interconnection of yeast models.

**Attractor**	**Boolean representation**	**$w_{A}^{I}$**
$A_{11*}^{1}$	00000000000	0.0802
$A_{11*}^{2}$	00000000100	0.0882
$A_{11*}^{3}$	00001000000	0.0792
$A_{11*}^{4}$	00001000100	0.0893
$A_{11*}^{5}$	00110000000	0.0669
$A_{11*}^{6}$	01000000100	0.0472
$A_{1*}^{7}$	01001000100	0.0490
$A_{2*}^{8}$	10110110011	0.0921
$A_{2*}^{9}$	11000111011	0.1290
$A_{2*}^{10}$	11110110011	0.0749
$A_{2*}^{11}$	11110111011	0.2039
**Attractor**	**Boolean representation**	$w_{A}^{I}$
$B_{12}^{1}$	10100110	0.5
$B_{21}^{2}$	11011001	0.5

Attractors and incidence probabilities, proportional to the size of each basin of attraction. For module Σ_B_, there exists a unique attractor for each v, hence $w_{B}^{1}=w_{B}^{2}$. The symbol * means that the output of this attractor depends on the function h^A^(a): we have $A_{12}^{4}$ with a G1 indicator but $A_{11}^{4}$ in the other two cases.

#### 5.2.2. Network interconnection, asymptotic and cross graphs

To establish a scheme of interconnection, observe that the cell size model acts on the cell cycle by triggering the start signal, that is START is given by (folded/free) protein *Cln*3*F*. Conversely, the input of the cell cycle to the cell size module is still unknown, the combination of variables and/or quantities used by the cell to detect its own size is a question for further analysis. As an hypothesis, we will assume that growth rate is detected through cell phase, since the cell cycle model provides this information. To explore the plausibility of this hypothesis, we will thus consider three different indicators of the cell cycle phase (M, S, and G1 phases) and compare the asymptotic graphs of the three corresponding interconnection schemes:

(13) $$\begin{array}{c} \text{M-phase}:u=h^{B}(b)=Cln3F,\;\;\;\upsilon =h^{A}(a)=Swi5\wedge Cdc20, \end{array}$$

(14) $$\begin{array}{c} \text{S-phase}:u=h^{B}(b)=Cln3F,\;\;\;\upsilon =h^{A}(a)=Clb5, \end{array}$$

(15) $$\begin{array}{c} \text{G1-phase}:u=h^{B}(b)=Cln3F,\;\;\;\upsilon =h^{A}(a)=Cdh1\wedge Sic. \end{array}$$

**Figure 6:**
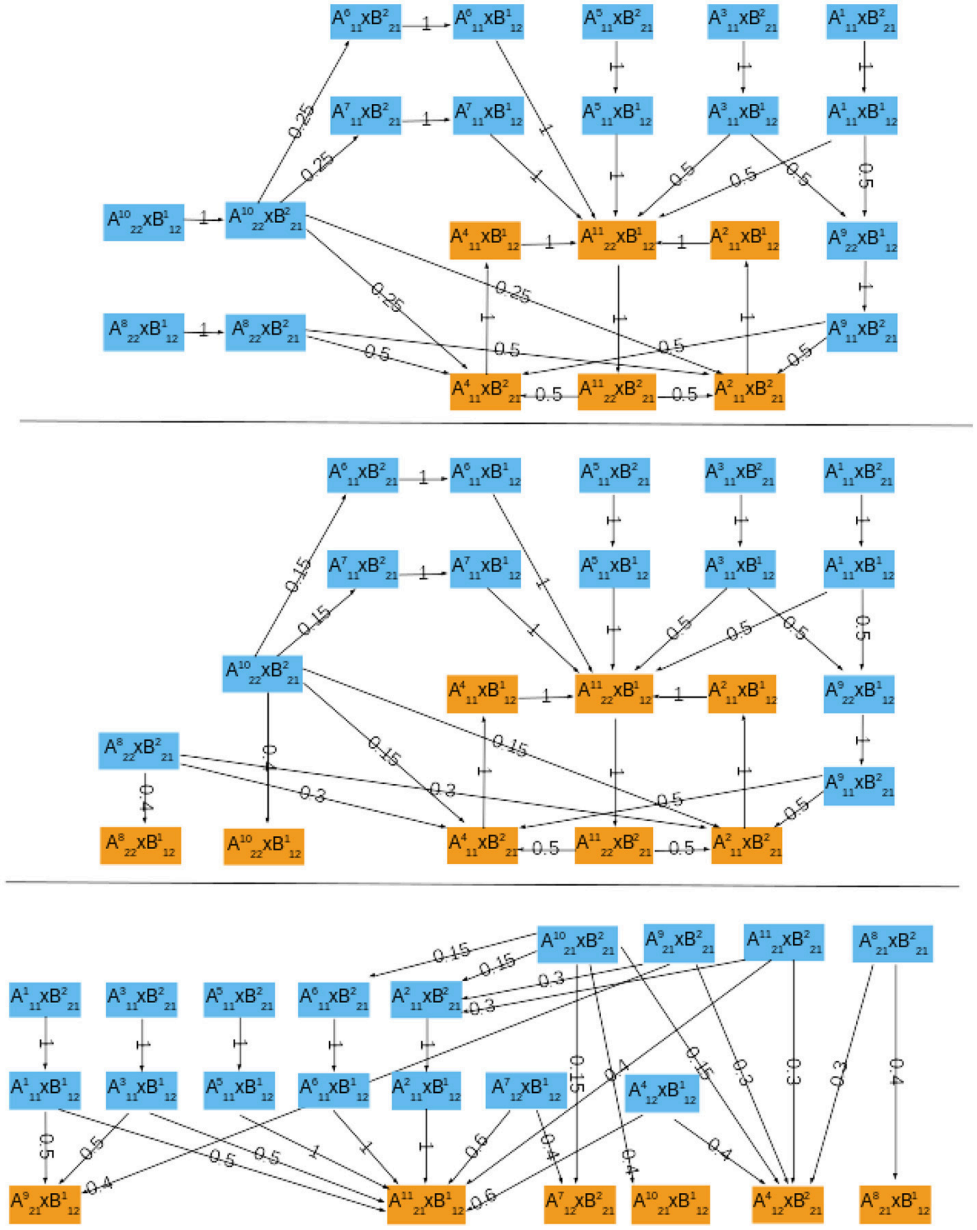
The probabilistic asymptotic graphs for the interconnected yeast network, with growth rate measured by different indicators of the cell cycle. Orange colored nodes belong to an attractor. **(Top)** M phase indicator, there is exactly one (cyclic) attractor. **(Middle)** S/G2 phase indicator, there are two single state attractors and one cyclic attractor. **(Bottom)** G1 phase indicator, there are six single state attractors.

In the case of growth measured by M phase (*h^A^*(*a*) = *Swi*5∧*Cdc*20), the asymptotic graph has a unique, cyclic, attractor (Figure 6, top):

$$\begin{array}{l} R_{1}^{M}=\{A_{22}^{11}\times B_{12}^{1},A_{22}^{11}\times B_{21}^{2},A_{11}^{2}\times B_{21}^{1},A_{11}^{2}\times B_{12}^{1},A_{11}^{4}\times \\ \;\;\;\;\;\;\;\;\;\;\;B_{21}^{2},A_{11}^{4}\times B_{12}^{1}\} \end{array}$$

This information is confirmed and complemented by computation of the cross graph, which has 524,288 nodes (= 2^19^). Attractor $R_{1}^{\text{M}}$ is composed of 116,520 states.

Interestingly, although neither Σ*^A^* nor Σ*^B^* have periodic orbits, in this case the interconnected network does exhibit an oscillatory orbit: at stationary G1 ($A_{11}^{4}$) the START signal ($B_{12}^{1}$) is received and the module Σ*^A^* performs one cell cycle:

$$A_{11}^{4}\times B_{21}^{2}\to A_{11}^{4}\times B_{12}^{1}\to A_{22}^{11}\times B_{12}^{1}\to A_{22}^{11}\times B_{21}^{2},$$

setting *Cln*3 back to its OFF state ($B_{21}^{2}$) and ending “near” M phase ($A_{22}^{11}$). At this point, the system returns to stationary G1 and repeats the cycle, waiting for cell to grow and again send the start signal. Two alternative paths are proposed for the cell cycle, with G1-phase described either by $A_{11}^{4}$ or similar state $A_{11}^{2}$. Since *G^as^* contains a unique attractor, its relative probability *P_rel_* is necessarily 1.

In the case of growth rate measured by S phase (*h^A^*(*a*) = *Clb*5), the asymptotic graph (Figure 6, middle) has three attractors, two single steady state and one cyclic attractor:

$$\begin{array}{l} R_{1}^{S}=\{A_{22}^{11}\times B_{12}^{1},A_{22}^{11}\times B_{21}^{2},A_{11}^{2}\times B_{21}^{1},\\ \;\;\;\;\;\;\;\;\;\;A_{11}^{2}\times B_{12}^{1},A_{11}^{4}\times B_{21}^{2},A_{11}^{4}\times B_{12}^{1}\}\\ R_{2}^{S}=\left\{A_{21}^{8}\times B_{12}^{1}\right\}\\ R_{3}^{S}=\left\{A_{21}^{10}\times B_{12}^{1}\right\} \end{array}$$

In this case, however, computation of the cross graph shows that $R_{1}^{S}$ is a spurious attractor, implying that the asymptotic graph has lost some information on transient pathways. In practice, the full graph contains pathways eventually leading from $R_{1}^{S}$ to either $R_{2}^{S}$ or $R_{3}^{S}$. This example shows the importance of verifying whether any of the asymptotic graph's attractors is spurious, and hence the usefulness of a complementary method as the cross graph. In this situation, the probabilistic interpretation of the asymptotic graph is unclear. The relative probabilities computed according to (8) yield equal probabilities for reaching attractors $R_{2}^{S}$ and $R_{3}^{S}$ (see Table 3). In contrast, $R_{1}^{S}$ must now be interpreted as a transient set of states.

**Table 3:** Attractors of the yeast interconnected system and their relative probabilities, *P_rel_*(*R_i_*), for different updating probabilities ϱ*_A_*.

**Case S-phase output**			
**Attractor**	**_*ϱ**A***_ = 0.2**	**_*ϱ**A***_ = 0.5**	**_*ϱ**A***_ = 0.7**
$A_{21}^{8}\times B_{12}^{1}$	0.1125	0.0938	0.0813
$A_{21}^{10}\times B_{12}^{1}$	0.1125	0.0938	0.0813
$R_{1}^{S}$	0.7750	0.8125	0.8375
**Case G1-phase output**			
**Attractor**	ϱ_***A***_ = 0.2	ϱ_***A***_ = 0.5	ϱ_***A***_ = 0.7
$A_{12}^{4}\times B_{21}^{2}$	0.1042	0.1266	0.1415
$A_{12}^{7}\times B_{21}^{2}$	0.0454	0.0401	0.0365
$A_{21}^{8}\times B_{12}^{1}$	0.0829	0.0691	0.0599
$A_{21}^{9}\times B_{12}^{1}$	0.1779	0.1585	0.1456
$A_{21}^{10}\times B_{12}^{1}$	0.0674	0.0562	0.0487
$A_{21}^{11}\times B_{12}^{1}$	0.5221	0.5495	0.5678

In the case G1 is used as measure of growth rate, we have *h^A^*(*a*) = *Cdh*1∧*Sic* and the asymptotic graph (Figure 6, bottom) has six single state attractors but *no cyclic attractor*:

$$\begin{array}{l} R_{1}^{G1}=A_{12}^{4}\times B_{21}^{2},\;R_{2}^{G1}=A_{12}^{7}\times B_{21}^{2},\\ R_{3}^{G1}=A_{21}^{8}\times B_{12}^{1},\;R_{4}^{G1}=A_{21}^{9}\times B_{12}^{1},\\ R_{5}^{G1}=A_{21}^{10}\times B_{12}^{1},\;R_{6}^{G1}=A_{21}^{11}\times B_{12}^{1}. \end{array}$$

All these attractors are confirmed by the cross graph. Computation of relative probabilities shows that the single steady state $A_{21}^{11}\times B_{12}^{1}$ is more frequently observed (with a percentage of around 54%, see Table 3). In this state all proteins of the cell cycle are expressed except for *Cdh*1 and *Sic*1, which characterize stationary G1 phase. The cell growth module is in a state where *Cln*3*F* is available, thus setting START to 1. The interconnected system is thus locked in a steady state where the interaction links are fixed: $A_{21}^{11}\times B_{12}^{1}$ = 11110111011 × 10100110, since the output of each attractor is equal to the input of the other.

#### 5.2.3. Hypotheses discrimination

These results appear to support a model for START signal of the form (12), as suggested by Aldea et al. (2017). Indeed, if cell size triggers START, then it can be assumed that there is a “critical size” which will be attained most probably at the end of G2 phase. And, in fact, the interconnected system exhibits an oscillatory cycle only in the case of M phase used as cell size indicator. This cycle is in agreement with cell cycle progression, meaning that the cell size module is able to trigger the START signal.

In contrast, when G1 or S phases are used as cell size indicator, the interconnected system has no oscillatory behavior. For the G1 case, the most frequent steady state ($A_{21}^{11}\times B_{12}^{1}$) represents a configuration where the cell size module permanently sets *Cln*3*F* = 1, and does not admit cell size to reset to zero. Note that G1 is the beginning of the cell cycle and a misleading indicator of “critical” size; in this case, the “critical” size is so small that the cell size module sets START permanently to 1 thus preventing the cell cycle to reset to zero and initiate a new cycle. Cells are locked in a steady state near mitosis and before early G1.

In conclusion, our analysis shows that neither *G*1 nor S phases are reliable cell growth indicators, but components from M phase are plausible candidates for detecting cell growth. We point out that the cell size Boolean network and the feedback interconnection points may admit several improvements, which are outside the scope of our paper. Nevertheless, we believe this first approach provides useful hints on how to further investigate and model the START signal in yeast.

## 6. Discussion and conclusions

Our work illustrates a new concept for the analysis of an interconnection of Boolean networks: the goal is to study the coupled behavior of two or more modules, using only the dynamics of each separate module. A new methodology has been discussed, based on construction of the asymptotic and cross graphs both representative of the full network transition graph and guaranteed to compute all attractors of the interconnected network. The two graphs have different properties but also complement each other. The cross graph provides exact results, in the sense that it contains all transient and asymptotic behaviors of the interconnected network. The asymptotic graph is a lighter construction containing a minimal number of nodes while recovering all attractors. In contrast to the cross graph, no bijection with the full network transition graph is guaranteed, implying that spurious attractors may appear; however, this happens at an extremely low rate (less than 1%).

Construction of the two graphs for random input/output networks with varying connectivity reveals their complementarity in terms of modules' connectivity: for low connectivity (*K_mean_* ≤ 5), the asymptotic graph is much smaller (on average 0.01% of the full graph, against 28% for the cross graph; Figure 2B) and faster to compute; in contrast, for high connectivity (*K_mean_* > 5), the size of the cross graph drastically reduces to 0.04% of the full graph (Figure 2A) becoming even faster to analyze than the asymptotic graph (Figure 2C). In addition, even though the asymptotic graph involves a drastic simplification of the state space, it has an unexpectedly high rate of accuracy, as shown in Figure 2D.

The practical advantages of our methodology are illustrated by the study of two well known biological networks. Among other useful characteristics, the asymptotic graph can greatly reduce the size of the state space, especially in the case of single-input single-output modules. For instance the mammalian and yeast interconnected networks, with an average connectivity of *K* = 2.76 and *K* = 2.68 respectively, have asymptotic graphs of only 18 and 22 nodes (compared to 2^17^ or 2^19^).

The analysis of the coupling between cell cycle and circadian clock shows that, according to experimental observations (for instance by Plikus et al., 2013), the asymptotic graph predicts that mammalian cells in the quiescent state may have a working clock. Furthermore, under general hypotheses, the probabilistic approach predicts that one third of cells are arrested in the quiescent state but still have circadian oscillations, while the other two thirds follow a normal cell cycle progression intertwined with circadian oscillations. In the budding yeast example, we have explored a recent hypothesis by Aldea et al. (2017) for a mechanism to trigger the START signal and initiate cell cycle. The mechanism is based on cell size detection through cell growth rate. Our analysis supports such a mechanism as a possible START trigger, and suggests that cell size indicator should come from an element during M phase.

The advantages of our analysis tools are multiple and particularly suited to the modeling of biological regulatory networks: by manipulating existing models as building blocks, the presented tools allow to rapidly simulate, compare, and test different coupling schemes or hypotheses on mutual regulatory effects, and therefore advance in the understanding of highly modular regulatory networks. The probabilistic interpretation and the analysis of transient behaviors emerge as two noteworthy directions for future developments in logical models.

## Author contributions

MC and LT: equally contributed to conception, analysis and design of the study; MC and LT: wrote and revised the manuscript.

### Conflict of interest statement

The authors declare that the research was conducted in the absence of any commercial or financial relationships that could be construed as a potential conflict of interest.

## Supplementary material

The Boolean models used for mammalian and budding yeast cell cycles are provided as Supplementary Material.

## Appendix

### Proof of theorem 2

Let G = (V, ε) be a digraph and let ν, ν′ ∈ V be any two vertices of G. Introduce the following notation:

- ν → G ν′ means that there is an edge from ν to ν′ in G, i.e., (ν, ν′) ∈ ε (ν′ is a *successor* of ν).
- ν ▹ G ν′ means that there exists a path from ν to ν′ in G, i.e., there exist *k* ≥ 0 vertices ν_1_, …, ν_*k*_ such that ν = ν_1_ → G ν_2_ → G… → *_G_* ν_*k*_ = ν′ (ν′ is a *descendant* of ν).
- ν ~ G ν′ means that there exists a path from ν to ν′ and a path from ν′ to ν in G (ν and ν′ are mutually reachable from each other). The relation ~ _G_ is an equivalence over V × V.

Remark that according to the definition of partition $Z_{h}^{A}$, any $A_{\alpha }^{i}$ is included in a SCC of each graph *G^A,u^*, in other words:

$$\forall a,a^{\prime }\in A_{a}^{i},\forall u\in {\left\{0,1\right\}}^{p_{A}},a{∼}_{G^{A,u}}a^{\prime }.$$

For convenience, we introduce the two following maps π and ψ, establishing relationships between the two vertex sets *V^cr^* and Ω = {0, 1}^*n_A_* + *n_B_*^.

- For *V* = $A_{\alpha }^{i}$ × $B_{\beta }^{j}$ ∈ *V^cr^*, let π(*V*): = {(*a, b*)|*a* ∈ $A_{\alpha }^{i}$, *b* ∈ $B_{\beta }^{j}$} ⊆ Ω; and for *Q* = {*V*_1_, …, *V_k_*} ⊆ *V^cr^*, define $\pi (Q):=\cup_{l=1}^{k}\pi (V_{l})\subseteq \Omega$.
- For *x* = (*a, b*) ∈ Ω, by definition of $Z_{h}^{A},Z_{h}^{B}$ there is a unique $A_{\alpha }^{i}$ and a unique $B_{\beta }^{j}$ such that $A_{\alpha }^{i}$ ∋ *a*, $B_{\beta }^{j}$ ∋ *b*. Let ψ(*x*): = $A_{\alpha }^{i}$ × $B_{\beta }^{j}$; by extension, for *S* ⊆ Ω, define ψ(*S*): = {ψ(*x*)|*x* ∈ *S*} ⊆ *V^cr^*.

Theorem 2 is a consequence of the two following lemmas.

lemma 1. *Let* *x, y* ∈ Ω *such that* *x* ▹ _*G*_ *y**, then either* ψ(*x*) = ψ(*y*)*, or* ψ(*x*) ▹ _*G^cr^*_ ψ (*y*).

proof. Suppose first that *x* → _*G*_ *y*, that is to say either (i): *x* = (*a, b*) → _*G*_ (*a′*, *b*) = *y* where *a* → _*G^A,h^B^(b)^*_ *a′* or (ii): *x* = (*a, b*) → _*G*_ (*a, b′*) = *y* where *b* → _*G^B,h^A^(a)^*_ *b′*. These two cases being perfectly symmetrical, consider for instance case (i). Let $A_{\alpha }^{i},A_{\alpha^{\prime }}^{i^{\prime }}$ and $B_{\beta }^{j}$ be respectively the (unique) sets such that $a\in A_{\alpha }^{i},a^{\prime }\in A_{\alpha^{\prime }}^{i^{\prime }}$ and $b\in B_{\beta }^{j}$. Two cases are to be considered.

Case 1: suppose $A_{\alpha }^{i}\neq A_{\alpha^{\prime }}^{i^{\prime }}$, then ψ(*x*) = $A_{\alpha }^{i}$ × $B_{\beta }^{j}$ = ψ(*y*).

Case 2: suppose $A_{\alpha }^{i}\neq A_{\alpha^{\prime }}^{i^{\prime }}$. Then according to the definition of *G^cr^*, from *a* → _*G^A,β^*_ *a′* we deduce that $\psi (x)=A_{\alpha }^{i}\times B_{\beta }^{j}\to_{G^{cr}}A_{\alpha^{\prime }}^{i^{\prime }}\times B_{\beta }^{j}=\psi (y)$.

Suppose now that *x* ▹ _*G*_ *y*, *ie.*, *x* = *x*_1_ → _*G*_ *x*_2_ → _*G*_ … → _*G*_ *x_k_* = *y*. By applying successively the previous result along that path, we deduce that either ψ(*x*) = ψ(*y*) or ψ(*x*) ▹ _*G^cr^*_ ψ (*y*), which concludes the proof.    □

lemma 1. *Let* *V, V′* ∈ *V^cr^* *be two vertices of the cross graph*.

- ∀*x, y* ∈ π(*V*), *x* ~ _*G*_ *y*.
- *If* *V* ▹ _*G^cr^*_ *V′**, then for all* *x* ∈ π(*V*) *and* *y* ∈ π(*V′*), *x* ▹ _*G*_ *y*.

proof. Let start with assertion *(i)*. Let *V* = $A_{\alpha }^{i}$ × $B_{\beta }^{j}$, *x* = (*a, b*) ∈ π(*V*) and *y* = (*a′*, *b′*) ∈ π(*V*). Since *a* and *a′* both belong to the same $A_{\alpha }^{i},a\sim_{G^{A,\beta }}a^{\prime }$. In the same way, *b* ~ _*G^B,α^*_ *b′*. From there it is easy to verify that (*a, b*) ~ _*G*_ (*a′*, *b*) ~ _*G*_ (*a′*, *b′*), so *x* ~ _*G*_ *y*. Let us prove the second assertion. Suppose first that *V* → _*G^cr^*_ *V′*. For instance, let $V=A_{\alpha }^{i}\times B_{\beta }^{j}$ and $V^{\prime }=A_{\alpha^{\prime }}^{i^{\prime }}\times B_{\beta }^{j}$ with $A_{\alpha }^{i}∋a_{1}\to_{G^{A,\beta }}a_{2}\in A_{\alpha^{\prime }}^{i^{\prime }}$ (the symmetrical case can be treated completely analogously). Let *x* = (*a, b*) ∈ π(*V*) and *y* = (*a′*, *b′*) ∈ π(*V′*). Since *a* and *a*_1_ both belong to the same $A_{\alpha }^{i}$, we have *a* ~ _*G^A,β^*_ *a*_1_. Similarly $a^{\prime },a_{2}\in A_{\alpha^{\prime }}^{i^{\prime }}$, implying *a′* ~ _*G^A,β^*_ *a*_2_. Therefore we have *a* ~ _*G^A,β^*_ *a*_1_ → _*G^A,β^*_ *a*_2_ ~ _*G^A,β^*_ *a′*, hence (*a, b*)▹_*G*_ (*a′*, *b*). Now, since *b* and *b′* both belong to the same $B_{\beta }^{j}$ *b* ~ _*G^B,α′^*_ *b′*, which proves that (*a′*, *b*)▹_*G*_ (*a′*, *b′*), therefore *x*▹ _*G*_ *y*.

Suppose now that *V* ▹ _*G^cr^*_ *V′*, *ie.*, *V* = *V*_1_ → _*G^cr^*_ *V*_2_ → _*G^cr^*_ … → _*G^cr^*_ *V_k_* = *V′*. By applying successively the previous result along that path, we deduce that *x*▹ _*G*_ *y* for any *x* ∈ π(*V*) and *y* ∈ π(*V′*).

Lemmas 1 and 2 establish an exact correspondence between the paths in *G* and the paths in *G^cr^*. The proof of the theorem becomes rather straightforward. Indeed, suppose *Q* = {*V*_1_, …, *V_k_*} is a SCC of *G^cr^*. Then Lemma 2 implies that π(*Q*) is included in a SCC *S* of *G*. Suppose now that π(*Q*) ⊊ *S*, *ie*. there exists *y* ∈ *S*\ π (*Q*) such that ψ(*y*) ∉ *Q*. For any *x* ∈ π(*V*) ⊂ *S*, we have *x* ~ _*G*_ *y* (with Lemma 1), implying ψ(*y*) ∈ *Q* which is a contradiction. Therefore, π(*Q*) = *S*. Reciprocally, suppose *S* ⊆ Ω is a SCC of *G*. Then lemma 1 implies that ψ(*S*) is included in a SCC *Q* of *G^cr^*. Lemma 2 further yields ψ(*S*) = *Q*. By using a similar kind of reasoning, it is easy to show that there is an exact one-to-one correspondence between the terminal SCCs of *G^cr^* and the attractors of *G*.
