# Supplementary material for: Analysis Tools for Interconnected Boolean Networks With Biological Applications
Source: Front Physiol. 2018 May 29;9:586. doi: 10.3389/fphys.2018.00586 (PMC5987301; doi:10.3389/fphys.2018.00586)
Supplement: Supplementary file 1 [file Table_1.PDF]

Supplementary Material: Analysis tools for interconnected Boolean networks with biological applications

BOOLEAN MODELS FOR MAMMALIAN AND BUDDING YEAST CELL CYCLE

The mammalian cell cycle model used in the paper is due to Fauré et al. (2006). It comprises 10 variables governed by the following Boolean rules:

$$\begin{aligned}
CycD^+ &= CycD \\
Rb^+ &= (\neg CycD \wedge \neg CycE \wedge \neg CycA \wedge \neg CycB) \vee (p27 \wedge \neg CycD \wedge \neg CycB) \\
E2F^+ &= (\neg Rb \wedge \neg CycA \wedge \neg CycB) \vee (p27 \wedge \neg Rb \wedge \neg CycB) \\
CycE^+ &= \neg u \wedge (E2F \wedge \neg Rb) \\
CycA^+ &= (E2F \wedge \neg Rb \wedge \neg Cdc20 \wedge \neg(Cdh1 \wedge Ubc)) \vee (CycA \wedge \neg Rb \wedge \neg Cdc20 \wedge \neg(Cdh1 \wedge Ubc)) \\
p27^+ &= \neg CycD \wedge \neg CycE \wedge \neg CycA \wedge \neg CycB \\
Cdc20^+ &= CycB \\
Cdh1^+ &= (\neg CycA \wedge \neg CycB) \vee Cdc20 \vee (p27 \wedge \neg CycB) \\
Ubc^+ &= \neg Cdh1 \vee (Cdh1 \wedge Ubc \wedge (Cdc20 \vee CycA \vee CycB)) \\
CycB^+ &= \neg Cdc20 \wedge \neg Cdh1
\end{aligned} \tag{S1}$$

The yeast cell cycle model used in the paper was developed by Li et al. (2004). The dynamics of its 11 variables (S_i) are defined by the following rule:

$$S_i^+ = \begin{cases} 1, & \sum_j a_{ij} S_j > 0 \\ 0, & \sum_j a_{ij} S_j < 0 \\ S_i, & \sum_j a_{ij} S_j = 0 \end{cases}$$

where

$$a_{ij} = \begin{cases} 1, & S_j \rightarrow S_i \\ -1, & S_j \dashv S_i. \end{cases}$$

Equivalently, the model can be written in the following logical form:

$$
\begin{aligned}
&START^{+} = u \\
&MBF^{+} = (\neg Cln3 \wedge (\neg Clb1 \wedge MBF)) \vee (Cln3 \wedge (\neg Clb1 \vee MBF)) \\
&SBF^{+} = (\neg Cln3 \wedge (\neg Clb1 \wedge SBF)) \vee (Cln3 \wedge (\neg Clb1 \vee SBF)) \\
&Cln1^{+} = SBF \\
&Cdh1^{+} = ((\neg Clb5 \wedge \neg Clb1) \wedge (\neg Clb5 \vee \neg Clb1)) \vee (aux5 \wedge (\neg Cln1 \wedge Cdc \wedge Cdh1)) \\
&Swi5^{+} = (Mcm \wedge (\neg Clb1 \vee Cdc)) \vee (\neg Mcm \wedge (\neg Clb1 \wedge Cdc)) \\
&Cdc^{+} = Mcm \vee Clb1 \\
&Clb5^{+} = ((\neg(MBF \vee Cdc) \vee (MBF \wedge Cdc)) \wedge (\neg Sic1 \wedge Clb5)) \vee ((\neg Cdc \wedge MBF) \wedge (\neg Sic1 \vee Clb5)) \\
&Sic1^{+} = (b101 \wedge a101) \vee (b102 \wedge a102) \vee (b103 \wedge a103) \\
&Clb1^{+} = (b111 \wedge a111) \vee (b112 \wedge a112) \vee (b113 \wedge a113) \\
&Mcm^{+} = Clb1 \vee Clb5
\end{aligned} \tag{S2}
$$

with the following auxiliary expressions:

$$
\begin{aligned}
aux5 &= (\neg Cln1 \wedge \neg Cdc \wedge Cdh1) \vee (\neg Cln1 \wedge Cdc \wedge \neg Cdh1) \vee (Cln1 \wedge Cdc \wedge Cdh1) \\
\\
a101 &= Cdc \wedge \neg Clb5 \wedge \neg Clb1 \\
a102 &= (\neg Cdc \wedge \neg Clb5 \wedge \neg Clb1) \vee (Cdc \wedge (\neg Clb5 \vee \neg Clb1)) \\
a103 &= Cdc \vee \neg Clb5 \vee \neg Clb1 \\
b101 &= (\neg Cln1 \wedge \neg Swi5 \wedge \neg Sic1) \vee (Cln1 \wedge \neg Swi5 \wedge Sic1) \vee (Cln1 \wedge Swi5 \wedge \neg Sic1) \\
b102 &= (\neg Cln1 \wedge \neg Swi5 \wedge Sic1) \vee (\neg Cln1 \wedge Swi5 \wedge \neg Sic1) \vee (Cln1 \wedge Swi5 \wedge Sic1) \\
b103 &= \neg Cln1 \wedge Swi5 \wedge Sic1 \\
\\
a111 &= Clb5 \wedge \neg Cdh1 \wedge \neg Cdc \\
a112 &= (\neg Clb5 \wedge \neg Cdh1 \wedge \neg Cdc) \vee (Clb5 \wedge (\neg Cdh1 \vee \neg Cdc)) \\
a113 &= Clb5 \vee \neg Cdh1 \vee \neg Cdc \\
b111 &= (\neg Sic1 \wedge \neg Mcm \wedge \neg Clb1) \vee (Sic1 \wedge \neg Mcm \wedge Clb1) \vee (Sic1 \wedge Mcm \wedge \neg Clb1) \\
b112 &= (\neg Sic1 \wedge \neg Mcm \wedge Clb1) \vee (\neg Sic1 \wedge Mcm \wedge \neg Clb1) \vee (Sic1 \wedge Mcm \wedge Clb1) \\
b113 &= \neg Sic1 \wedge Mcm \wedge Clb1
\end{aligned}
$$

REFERENCES

Fauré, A., Naldi, A., Chaouiya, C., and Thieffry, D. (2006). Dynamical analysis of a generic boolean model for the control of the mammalian cell cycle. *Bioinformatics* 22, 124–131

Li, F., Long, T., Lu, Y., Ouyang, Q., and Tang, C. (2004). The yeast cell cycle is robustly designed. *PNAS* 101, 4781–4786
